# Proton-Conducting Membranes from Polyphenylenes Containing Armstrong’s Acid

**DOI:** 10.1021/acs.macromol.3c02123

**Published:** 2024-01-30

**Authors:** Andy Künzel-Tenner, Christoph Kirsch, Oleksandr Dolynchuk, Leonard Rößner, Maxime Wach, Fabian Kempe, Thomas von Unwerth, Albena Lederer, Daniel Sebastiani, Marc Armbrüster, Michael Sommer

**Affiliations:** † Institut für Chemie, Polymerchemie, Technische Universität Chemnitz, Straße der Nationen 62, 09111 Chemnitz, Germany; ‡ Institut für Chemie, Theoretische Chemie, Martin-Luther-Universität Halle-Wittenberg, Von-Danckelmann-Platz 4, 06120 Halle, Germany; § Experimental Polymer Physics, Martin Luther University Halle-Wittenberg, Von-Danckelmann-Platz 3, 06120 Halle, Germany; ∥ Institut für Chemie, Materialien für Innovative Energiekonzepte, Technische Universität Chemnitz, Straße der Nationen 62, 09111 Chemnitz, Germany; ⊥ Institut für Automobilforschung, Technische Universität Chemnitz, Reichenhainer Straße 70, 09126 Chemnitz, Germany; # Leibniz Institut für Polymerforschung Dresden e. V., Hohe Straße 6, 01069 Dresden, Germany; ¶ Department of Chemistry and Polymer Science, Stellenbosch University, Private Bag X1, 7602 Matieland, South Africa; ∇ Forschungszentrum MAIN, TU Chemnitz, Rosenbergstraße 6, 09126 Chemnitz, Germany

## Abstract

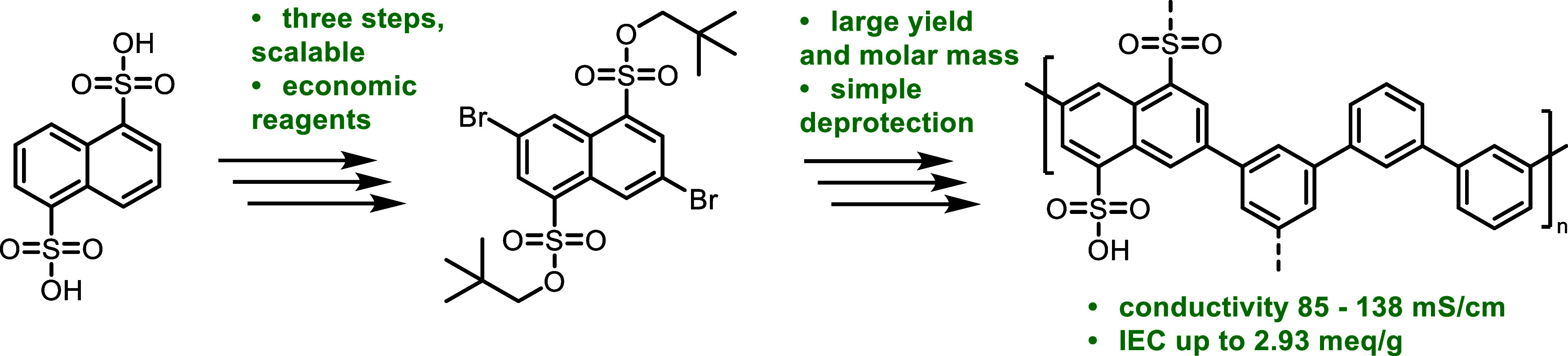

This study demonstrates the use of 1,5-naphthalenedisulfonic acid as a suitable building block for the efficient and economic preparation of alternating sulfonated polyphenylenes with high ion-exchange capacity (IEC) via Suzuki polycondensation. Key to large molar masses is the use of an all-*meta*-terphenyl comonomer instead of *m*-phenyl, the latter giving low molar masses and brittle materials. A protection/deprotection strategy for base-stable neopentyl sulfonates is successfully implemented to improve the solubility and molar mass of the polymers. Solution-based deprotection of polyphenylene neopentyl sulfonates at 150 °C in dimethylacetamide eliminates isopentylene quantitatively, resulting in membranes with high IEC (2.93 mequiv/g) and high proton conductivity (σ = 138 mS/cm). Water solubility of these copolymers with high IEC requires thermal cross-linking to prevent their dissolution under operating conditions. By balancing the temperature and time of the cross-linking process, water uptake can be restricted to 50 wt %, retaining an IEC of 2.33 mequiv/g and a conductivity of 85 mS/cm. Chemical stability is addressed by treatment of the membranes under Fenton’s conditions and by considering barrier heights for desulfonation using density functional theory (DFT) calculations. The DFT results suggest that 1,5-disulfonated naphthalenes are at least as stable as sulfonated polyphenylenes against desulfonation.

## Introduction

Proton-exchange membranes (PEMs) can be used in diverse applications. Besides their most prominent use in fuel cells^1−3^ as an ion-selective layer separating the two half cells, they can be utilized in electrolysis,^4,5^ water treatment,^6,7^ and redox-flow batteries.^8−10^ Material requirements for PEMs range from performance to stability, straightforward and reproducible preparation to economy of components, and finally aspects of recycling. Especially, thermal, mechanical, and chemical stabilities are a necessity, as well as a sufficiently high proton conductivity, usually considered to be σ > 0.1 S·cm^–1^.^1^ The benchmark is commercially available Nafion and derivatives, which are perfluorinated sulfonic acid (PFSA)-based membranes that fulfill a large portion of the requirements mentioned. Downsides are limited mechanical properties at elevated temperatures, high water and methanol crossover, and high costs.^11−14^ A further drawback is the environmental persistence of low molecular weight PFSA chemicals, which are also used for the synthesis of fluoropolymers and therefore render PFSA-based membranes problematic as well.^15,16^ The extremely high chemical stability of perfluorinated compounds allows for their worldwide distribution and finally enrichment in larger organisms, including humans, where negative health effects are just at the beginning to be investigated and understood.^15^

Major research efforts directed to fluorine-free alternatives have been made over the years, including sulfonated aromatic polyether ether ketones (SPEEKs), polysulfones (SPUs) polybenzimidazoles, polyimides, and others.^17−31^ Recently, sulfonated polyphenylenes with exclusive carbon–carbon bonds in the backbone have gained attention as synthetic routes are more versatile compared to SPEEK and SPU, thus allowing the implementation of a larger degree of modulation of chemical structure. Polyphenylenes have shown high chemical stability, excellent thermal and mechanical stabilities, and high proton conductivity.^32−37^ Examples include polyphenylenes made in three steps by Yamamoto coupling and in seven steps using Diels–Alder chemistry, yielding high chemical stability, ion-exchange capacity (IEC), and proton conductivity.^34,35,38,39^ However, further increasing ease and modularity of the synthetic route, reducing the number of reaction steps and costs of starting materials, and increasing the overall yield of the reaction sequence are all important to advance the ever-growing field of membrane-exchange polymers.

Here, we present a straightforward synthetic alternative to make alternating sulfonated polyphenylenes via Suzuki polycondensation (SPC). We use a neopentyl-protected form of 1,5-naphthalenedisulfonic acid, referred to as neopentyl-protected Armstrong’s acid (AA-NP), as a cost-economic, readily available building block with high sulfonic acid group density and copolymerize it with an all-*meta*-terphenyl comonomer *m*TP. *m*TP imparts mechanical toughness and further discriminates the copolymers from earlier reports of sulfonated polyphenylenes made by SPC.^40^ Notably, the presence of naphthalene instead of phenyl units in the polymer main chain is expected to alter the physical properties. Using the comparison of polyethylene terephthalate versus polyethylene naphthalate (PEN) as an example, the geometrical difference of the naphthalene units in PEN alters single-chain dimensions, which finally has an effect on, e.g., the glass-transition temperature.^41^ This, in turn, is a relevant property for membranes operated at elevated temperatures. Furthermore, π–π interactions between the naphthalene units of different chain segments have been reported to enhance the properties of proton-conducting polymers, namely, proton conductivity.^42^

The herein prepared protected copolymers P(AA-NP-*alt*-*m*TP) exhibit number-average molecular weights larger than *M*_n_ ∼ 60 kg/mol and can be dissolved in common nonpolar and polar aprotic solvents, which facilitates handling and workup. Deprotection in dimethylacetamide (DMAc) solution furnishes P(AA-*alt*-*m*TP) with free sulfonic acid groups. The high density of sulfonic acid groups causes a preferably high IEC of 2.93 mequiv/g but also renders P(AA-*alt*-*m*TP) water soluble. Thermal cross-linking is therefore required. For an optimized cross-linking time and temperature, water uptake (WU) can be restricted to 50 wt %, and IEC and proton conductivity can be maintained at 2.33 mequiv/g and 85 mS/cm, respectively. Density functional theory (DFT) calculations conducted on model compounds suggest barrier heights for desulfonation, indicating that naphthalene-1,5-disulfonic acid is at least as stable as singly sulfonated phenylenes.

## Results and Discussion

### Synthesis of **P(AA-NP-*alt*-*m*TP)**

**Scheme 1:**
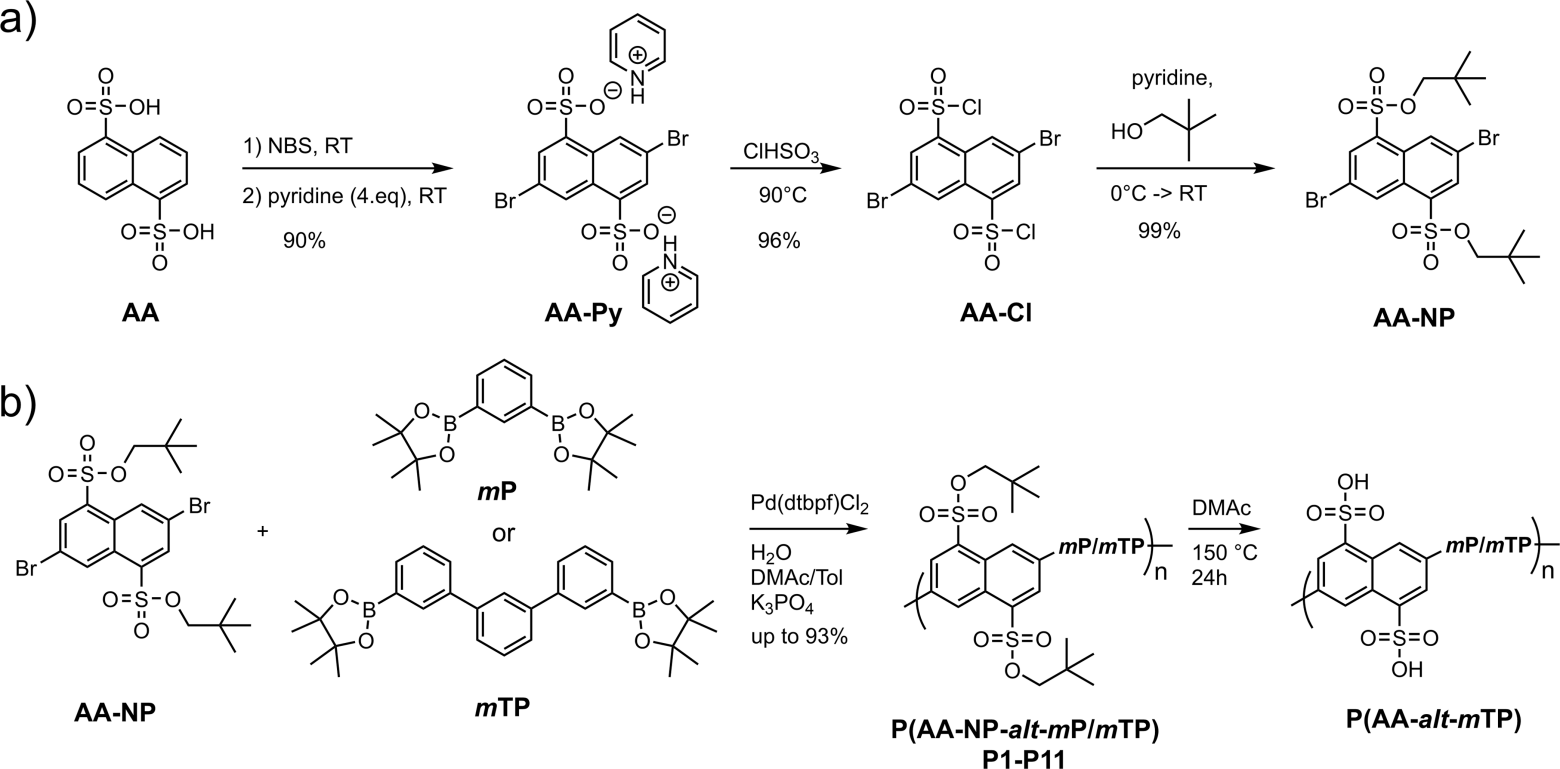
(a) Synthesis of Sulfonated Monomer AA-NP Based on Armstrong’s Acid and (b) SPC of AA-NP with *meta*-Phenylene Comonomers NBS: *N*-bromosuccinimide, DMAc: dimethylacetamide.

**Figure 1:**
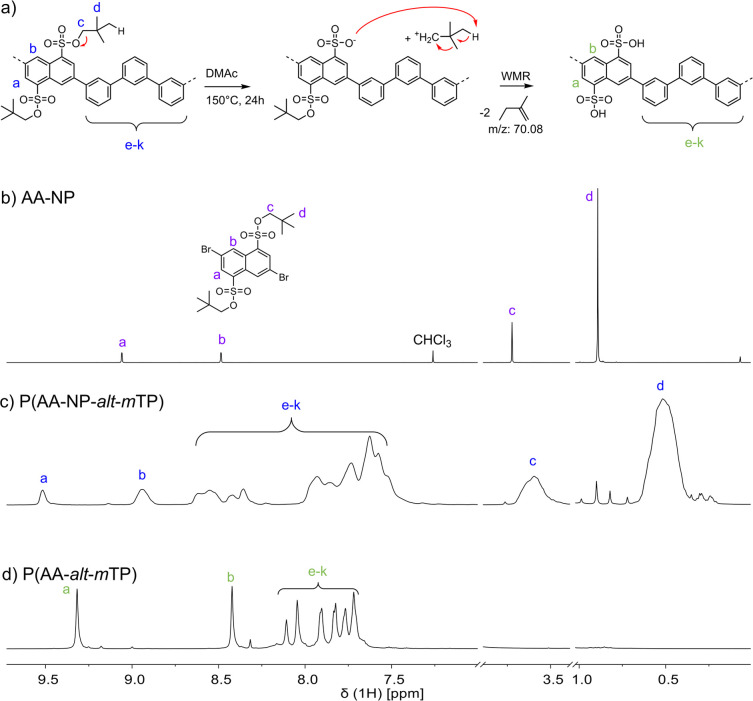
(a) Proposed mechanism of the deprotection of neopentyl sulfonates. (b) ^1^H NMR spectrum of AA-NP, (c) ^1^H NMR spectrum of protected P(AA-NP-*alt*-*m*TP), and (d) ^1^H NMR spectrum of deprotected P(AA-*alt*-*m*TP). WMR: Wagner–Meerwein rearrangement.

In order to establish a simple, yet modular polyphenylene with high and tunable IEC, we selected 1,5-naphthalenedisulfonic acid (“Armstrong’s acid”, referred to as AA) as a sulfonated building block. According to price estimations of the U.S. Department of Energy (DoE),^43^ cost-economic monomers and few, scalable reaction steps are desirable. Using AA as a readily available starting material, easily scalable bromination offers AA-based building blocks for various cross-coupling schemes. AA was brominated using *N*-bromosuccinimide (NBS) and precipitated from ethanol with pyridine to give dipyridinium salt AA-Py with an isolated yield of 90%. Chlorosulfonation furnished the corresponding sulfonyl chloride, AA-Cl, in 96% yield. Esterification with neopentyl alcohol delivered monomer AA-NP in quantitative yield (Scheme 1a, see also Figure 1b). While reports on the direct polymerization of charged, sulfonated building blocks via SPC have been presented,^44^ we have chosen to use protected sulfonates to facilitate polymerization and improve solubility in aprotic solvents. Protection of sulfonic acid with neopentyl groups was chosen due to their high stability under basic conditions required to sustain SPC conditions and to retain the possibility for later deprotection.^45^Scheme 1b summarizes all polymerizations. The chosen protocol of SPC allows for efficient preparation of polyphenylenes as well as simple handling of nontoxic boronic acid esters.^46^ To incorporate AA-NP into polyphenylenes, the comonomer of choice was *meta*-substituted phenyl comonomer *m*P. However, diverse polar solvents such as tetrahydrofuran (THF) or dioxane were not able to dissolve the resulting polymer. Therefore, solvent mixtures of DMAc, toluene (tol), and deionized water were chosen as reaction medium for polymerization. A volume ratio of 3.5:3.5:1 DMAc/tol/H_2_O was the best mixture that we were able to identify. Still, copolymers of AA-NP and *m*P, P(AA-NP-*alt*-*m*P) P1–5, exhibited limited solubility, seen by the formation of precipitates during polymerization that could not be redissolved in a variety of solvents such as CHCl_3_, THF, dimethylformamide, DMAc, or toluene, even at elevated temperature. The soluble fractions had all similarly low molecular weight materials (Table 1, entries 1–5) and exhibited very poor mechanical properties, i.e., films were very brittle. Therefore, *m*P was replaced by *meta*-terphenyl *m*TP, leading to significantly increased solubility and much larger molecular weights of P(AA-NP-*alt*-*m*TP) P6–11 (Table 1, entries 6–11). A deviation from the initially optimized solvent mixture did not further improve molar mass; hence, DMAc/tol/H_2_O 3.5:3.5:1 was used as well (Table 1, entries 6 and 7).

**Table 1:** Overview of Polymerizations of AA-NP with *m*P or *m*TP

entry	comonomer	equiv comonomer	solvent ratio DMAc/tol/H_2_O	*M*_w_ [kg/mol]	*M*_n_ [kg/mol]	*D̵*	yield [%]
P1^a^	*m*P	1.03	3.5:3.5:1	8.0	4.3	1.86	85
P2^a^	*m*P	1.04	3.5:3.5:1	19.7	16.2	1.22	88
P3^a^	*m*P	1.05	3.5:3.5:1	2.7	1.1	2.45	88
P4^a^	*m*P	1.06	3.5:3.5:1	35.4	26.2	1.35	96
P5^a^	*m*P	1.065	3.5:3.5:1	29.5	22.9	1.29	96
P6	*m*TP	1.045	2.5:2.5:1	73.4	49.0	1.5	62
P7	*m*TP	1.045	3:3:1	87.3	63.6	1.37	63
P8	*m*TP	1.045	3.5:3.5:1	102.3	51.8	1.97	93
P9	*m*TP	1.03	3.5:3.5:1	321.3	34.3	9.37	80
P10	*m*TP	1.047	3.5:3.5:1	88.9	56.2	1.58	91
P11	*m*TP	1.05	3.5:3.5:1	206.3	29.9	6.9	85

a Precipitate observed during polymerization, *M*_w_ and *M*_n_ from the dissolved fraction. All entries were carried out with K_3_PO_4_ (6 equiv) as the base and Pd(dtbpf)Cl_2_ (2 mol %) as the catalyst at 90 °C for 3 days.

To further optimize the molar mass and yield of P(AA-NP-*alt*-*m*TP), the equivalents of *m*TP were optimized. It is well known that subtle deviations from a 1:1 stoichiometry can have a huge impact on the molar mass of the resulting polymer.^46−48^ Since SPC is a step-growth-type polymerization with protodeborylation,^49^ oxidative deborylation or homocoupling^50^ being common side reactions that may occur to varying extent, it may be necessary to use a slight excess of boronic acid ester to ensure an effective 1:1 ratio of the relevant functional groups. It was found that a ratio of AA-NP to *m*TP (1:1.045) led to the highest molar mass and yield (Table 1, entry P8, *M*_w_ = 102.3 kg/mol, *M*_n_ = 51.8 kg/mol, and *D̵* = 1.97, 93%). The successful polymerization and structure of alternating copolymers P(AA-NP-*alt*-*m*TP) is shown by ^1^H NMR spectroscopy [Figure 1c, see also the Supporting Information for nuclear magnetic resonance (NMR) spectra and size exclusion chromatography (SEC) curves].

### Deprotection of **P(AA-NP-*alt*-*m*TP)**

**Figure 2:**
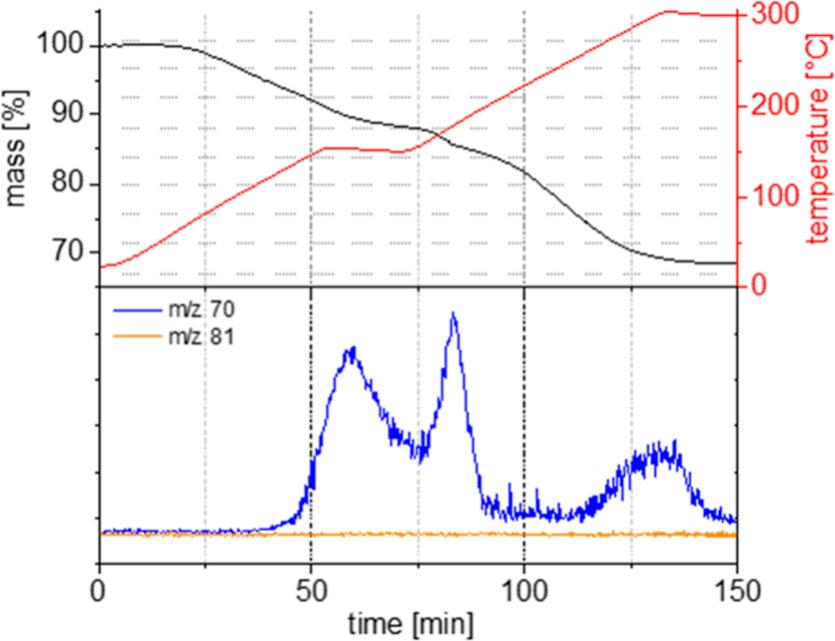
Thermal deprotection of P(AA-NP-*alt*-*m*TP) to obtain P(AA-*alt*-*m*TP) was investigated by TGA–MS.

To convert P(AA-NP-*alt*-*m*TP) into the acidic form, P(AA-*alt*-*m*TP), thermal bulk protocols as well as solution-based ones were compared to find the most simple yet efficient protocol for membrane formation. Several deprotection methods for sulfonate esters are known from the literature, including nucleophilic substitution via sodium azide^51^ and solid-state thermolysis at 150 °C.^52^ Because of the simplicity of the latter procedure, we first attempted to couple membrane casting and drying with deprotection. To obtain information about the progress of deprotection with time and temperature, thermogravimetric analysis coupled to mass spectrometry (TGA–MS) measurements were used with an isothermal period at 150 °C for 10 min. We anticipated that at this temperature, neopentyl groups were cleaved, while the sulfonic acid groups would remain stable. Thus, the *m*/*z* ratios of 70 and 81 were probed, corresponding to the formation of isopentylene and sulfurous acid (H_2_SO_3_), respectively (Figure 2).

From this measurement, the sulfonic acid ester groups appeared to be stable during the temperature range probed (Figure 2 bottom, orange line). However, isopentylene formation started at approximately 120 °C, continued during the isothermal period at 150 °C, and further extended to temperatures of ∼200 °C (Figure 2 bottom, blue line). Surprisingly, the *m*/*z* trace of isopentylene showed a second broad signal between ∼200 and 300 °C, indicating that deprotection was incomplete during the lower temperature range. Possible reasons may be related to surface area and morphology effects observed in bulk materials.

With thermal protection being less straightforward, we aimed for a quantitative, simple, and economic approach. Dissolving P(AA-NP-*alt*-*m*TP) in DMAc followed by stirring at 150 °C for 24 h allowed for quantitative deprotection. Figure 1c,d shows the comparison of the ^1^H NMR spectra of protected P(AA-NP-*alt*-*m*TP) and deprotected P(AA-*alt*-*m*TP), revealing complete conversion of the sulfonate ester to sulfonic acid. The observation of gas evolution during the reaction supported the assumption of the elimination of isopentylene. Based on these results, we suggest that degradation of the neopentyl ester occurs by heterolytic cleavage of the O–C– bond, leading to a primary carbenium ion, followed by Wagner–Meerwein rearrangement and reprotonation to yield isopentylene and sulfonic acid (Figure 1a).

### Morphology of P(AA-*alt*-*m*TP)

**Figure 3:**
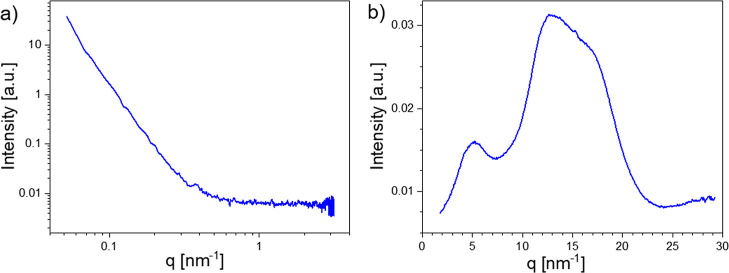
SAXS (a) and WAXS (b) curves of P(AA-*alt*-*m*TP) (sample P8 measured at room temperature under vacuum).

To investigate the ordering and morphology in P(AA-*alt*-*m*TP), small-angle X-ray scattering (SAXS) and wide-angle X-ray scattering (WAXS) measurements were performed (Figure 3). The featureless SAXS curve in Figure 3a suggests that there is no periodic microphase separation in the sample. In contrast, the WAXS curve in Figure 3b exhibits three broad peaks, two of which overlap to form a broad asymmetric scattering signal in the *q* range of 10–20 nm^–1^. These three peaks correspond to periodic spacings of about 1.2, 0.5, and 0.36 nm, respectively. The large peak width, exceeding that of typical crystal reflections, indicates that the observed WAXS scattering signals represent only short-range ordering. Thus, from the WAXS and SAXS results, it can be concluded that only small aggregates are formed in the sample, with no large-scale periodic structures due to microphase separation.

### Thermal Cross-Linking and Mechanical Properties of P(AA-*alt*-*m*TP)

**Figure 4:**
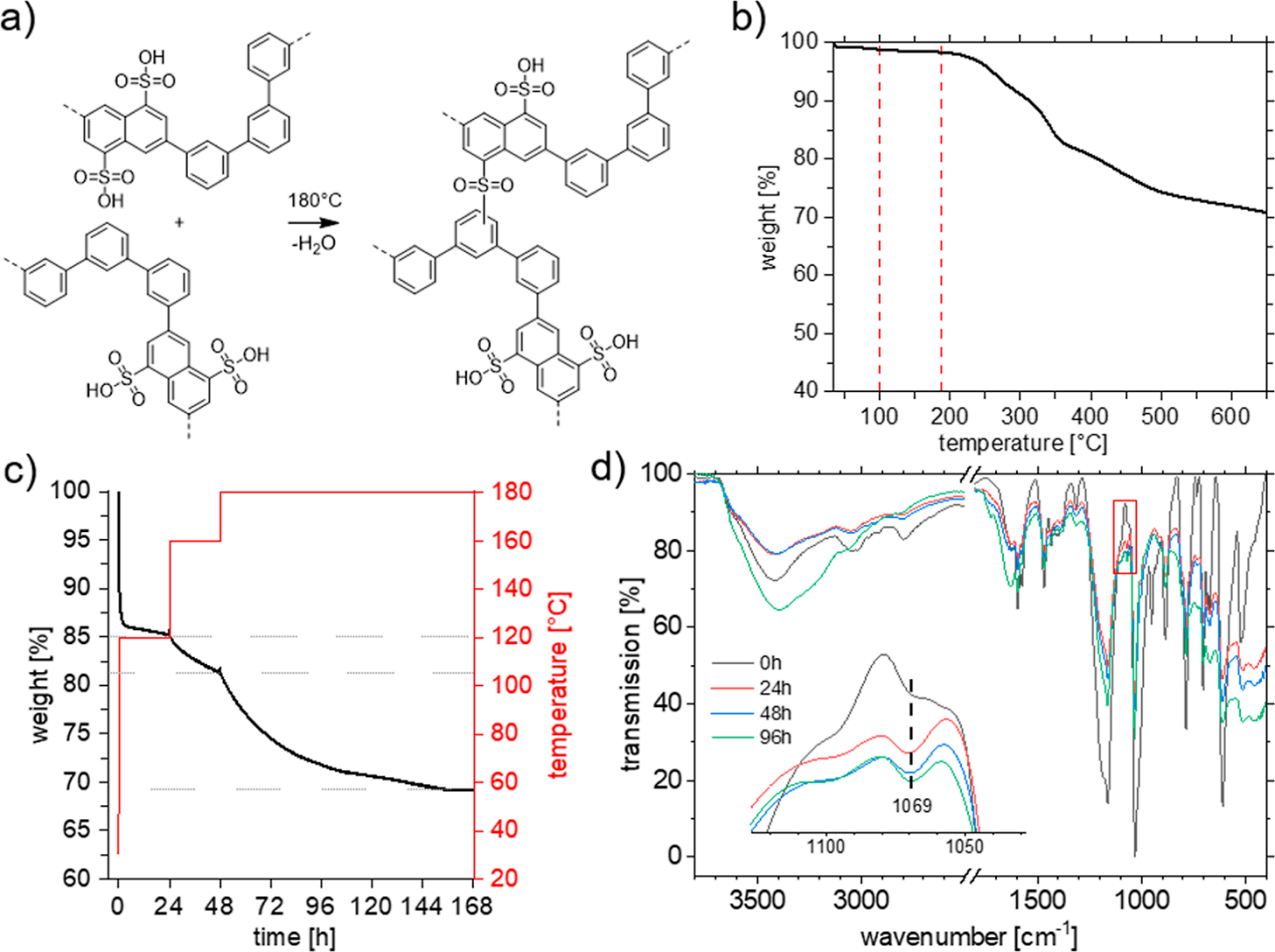
(a) Proposed sulfone formation during cross-linking. Note that the position of the linkage is unselective. (b) Thermogravimetry of deprotected P(AA-*alt*-*m*TP) under an inert atmosphere and 10 K/min. The red dashed lines mark the temperature of the first mass loss (100 °C) and the temperature for cross-linking (*T* = 180 °C). (c) Thermogravimetry of deprotected P(AA*-alt*-*m*TP) with stepwise isothermal phases at 120, 160, and 180 °C. (d) Comparison of IR spectra of P(AA-*alt*-*m*TP) after different times of cross-linking at 180 °C. The inset shows the decreasing intensity of transmission at 1069 cm^–1^ with increasing cross-linking time, which we ascribe to sulfone formation.

From thus prepared P(AA-*alt*-*m*TP), membranes were cast from DMAc, redissolved in DMSO, and cast again prior to cross-linking, yielding flexible and transparent films. Using DMSO as a casting agent prior to cross-linking resulted in improved results.^53^ However, the films were well soluble in water, suggesting that cross-linking is required to prevent unlimited WU and finally dissolution. In order to simplify the procedure, we anticipated that thermal treatment of the deprotected P(AA-*alt*-*m*TP) would lead to cross-linking in situ by virtue of the reaction of sulfonic acids group with the *m*TP comonomer units (Figure 4a).^54^ Note that the position of cross-linking is likely unselective with respect to both the phenyl ring and the carbon atom. To select appropriate temperatures for cross-linking, films of P(AA-*alt*-*m*TP) were subjected to further TGA experiments under standard as well as temperature- and time-dependent conditions under an inert atmosphere and 10 K/min heating rate (Figure 4b,c).

Figure 4b displays several mass losses. The first one at ∼100 °C was ascribed to the loss of water, which could not be removed during drying of the membrane. A second weight loss step (27.3%) at ∼180 °C (see dashed red lines) was attributed to the onset of the desulfonation reaction, suggesting sufficient thermal stability at least for low-temperature-range fuel cell applications.^55^ This degradation temperature is considerably high,^39,56^ but one needs to bear in mind that rate-dependent measurements may be entirely different from isothermal ones taken at temperatures of interest.^57^ Therefore, fine-tuning of the cross-linking process was obtained from further TGA measurements, including isothermal steps. Figure 4c shows the thermal degradation of a P(AA-*alt*-*m*TP) membrane over 1 week, starting stepwise at 120 °C for 24 h, 160 °C for 24 h, and finally 180 °C for 5 d. While after the initial evaporation of water, mass loss is negligible at 120 °C, a weight loss of ∼4% (with respect to the initial mass) was observed at 160 °C. A further 12 wt % mass loss occurred at 180 °C over a period of 5 d (Figure 4c). Thus, it is anticipated that cross-linking may start as early as 160 °C but is practically carried out at 180 °C. Differential scanning calorimetry (DSC) of P(AA-*alt*-*m*TP) in the temperature range 30–210 °C showed an endothermic broad signal between 30 and 180 °C, whose origin is attributed to restrained water within the polymer, as demonstrated for other polymers.^58^ From these measurements, a glass-transition temperature, *T*_g_, could not be extracted, but can be considered sufficiently high on the basis of the chemical structure with only sulfonic acids as side chains (see Figure S23).

The cross-linking process and formation of sulfone linkages between P(AA*-alt*-*m*TP) chains were further monitored using NMR and IR spectroscopies. Di Vona et al. obtained detailed ^13^C NMR spectra of SPEEK in DMSO before and after cross-linking,^59^ which was not possible for the herein investigated cross-linked P(AA*-alt*-*m*TP). Solid-state NMR was attempted, but visible changes in the ^13^C NMR spectra upon cross-linking were absent (Figure S24). Instead, an inspection of the IR spectra appeared to be more appropriate (Figure 4d). Several changes at different wave numbers could be seen; however, the clearest trend was a decreasing transmission with increasing cross-linking time at 1069 cm^–1^ (see the inset of Figure 4d). We ascribe this wavenumber to sulfone linkage formation according to Di Vona et al., who assigned a wavenumber of 1065 cm^–1^ to sulfone linkages in SPEEK.^53^

**Figure 5:**
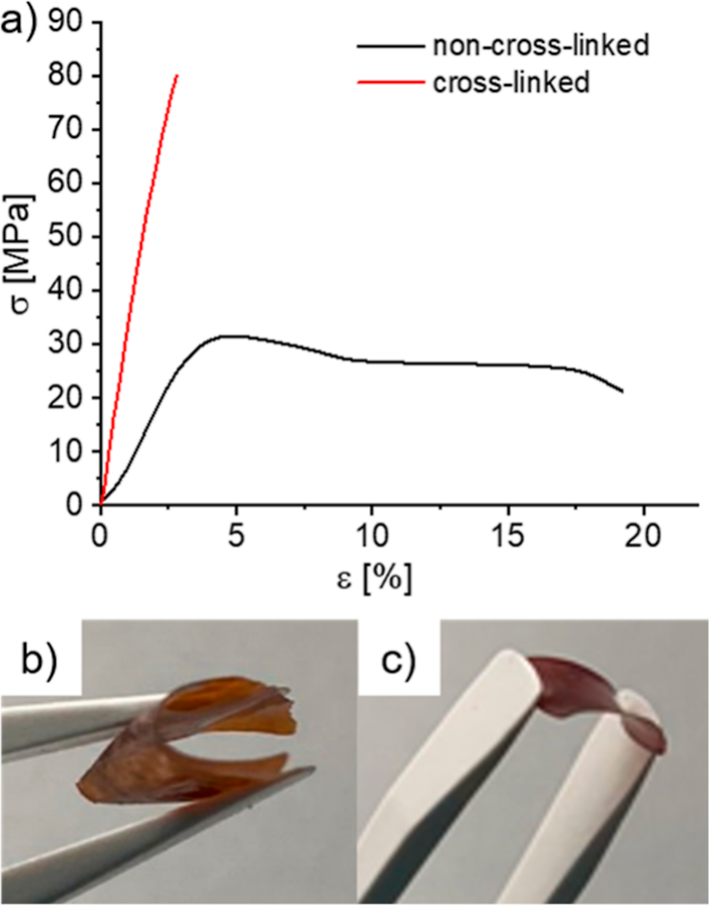
(a) Stress–strain experiment of cross-linked and non-cross-linked P(AA-*alt*-*m*TP) in the dry state. (b) Images of a non-cross-linked (b) and at 180 °C for 24 h cross-linked (c) membranes of P(AA-*alt*-*m*TP).

Furthermore, stress–strain experiments were conducted to investigate the mechanical properties of P(AA-*alt*-*m*TP) in the dry state before and after cross-linking (Figure 5a). While the non-cross-linked material (Figure 5b) showed typical behavior of a moderately ductile material with a strain at break of 19% and a yield strength of 31 MPa, membranes became much stiffer after cross-linking at 180 °C for 24 h with an increased Young’s modulus and a stress at break of 80 MPa, however at the cost of plastic deformation. Additional increases in the cross-linking time led to significantly more brittle membranes that easily broke upon bending (Figure 5c), suggesting further room for improvement. Yet, membranes could be handled in their wet states for further investigation of IEC, WU, and ionic conductivity as a function of cross-linking time.

### IEC, WU, and Proton Conductivity

**Figure 6:**
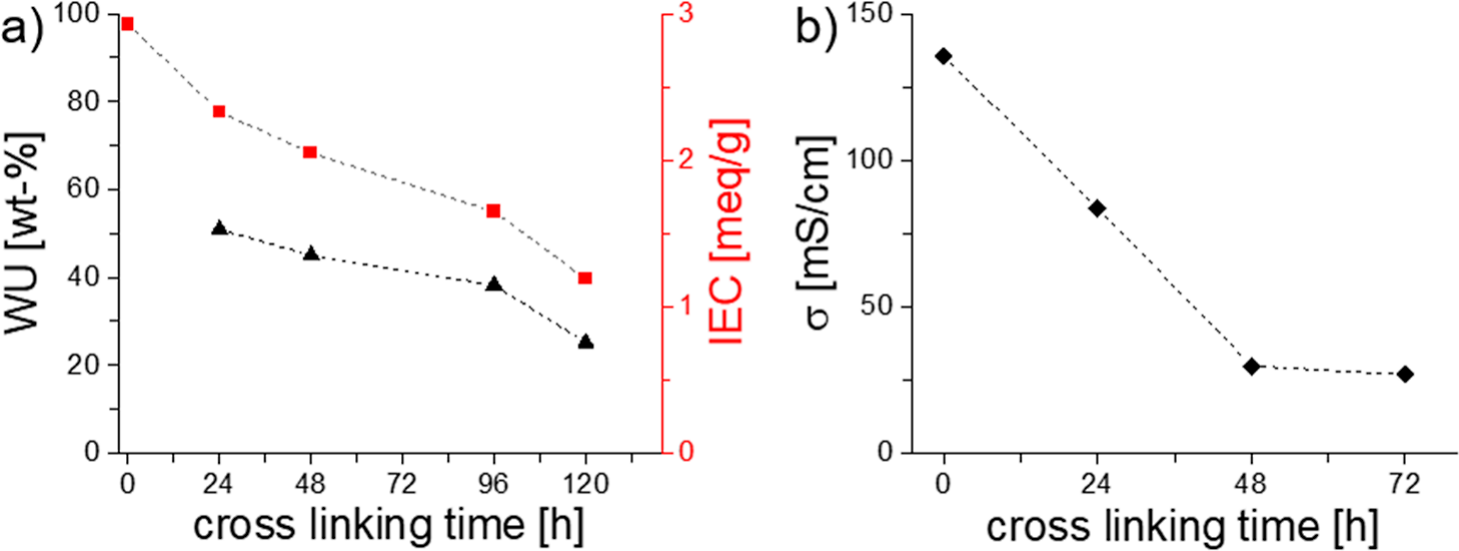
(a) WU and IEC of thermally cross-linked P(AA-*alt-m*TP) as a function of the duration of cross-linking. (b) Proton conductivity of thermally cross-linked P(AA-*alt-m*TP) as a function of the duration of cross-linking.

Figure 6a shows the IEC as determined via titration as well as the WU. As expected, the non-cross-linked material with the largest amount of sulfonic acid groups exhibited the highest IEC of 2.93 mequiv/g, which decreased with prolonged time of cross-linking. It is however noteworthy that after 24 h of cross-linking, a high IEC value of 2.33 mequiv/g was retained, while the WU strongly reduced to 50 wt %. The non-cross-linked material was soluble in water, excluding the determination of WU. For cross-linked membranes, the WU correlated with cross-linking time. A cross-linking time of 24 h reduced the WU to 50 wt %; longer times led to continuously decreased WU values down to 23%. This is congruent with the results from IR spectroscopy that suggest a denser and stiffer network for longer cross-linking times that come along with reduced WU.

Proton conductivities were measured in through-plane geometry as a function of the cross-linking time (Figure 6b). It is important to note that in order to obtain reproducible results with reasonable trends, cross-linking in an inert atmosphere was required. The non-cross-linked material showed a high proton conductivity of 138 mS/cm, which was lower compared to membrane materials based on sulfonated polyphenylenes,^32^ but still in a range suitable for fuel cell operation, where a value >100 mS/cm is considered a threshold value.^20^ With increasing cross-linking time, proton conductivity decreased in a controlled way, allowing to balance conductivity and WU. The decrease in σ with increasing cross-linking time is caused by the lower amount of sulfonic acid groups available and the resulting reduced WU. After 24 h of cross-linking, the conductivity reached 85 mS/cm and further decreased for longer times. Yet, for a cross-linking time of 24 h, P(AA-*alt-m*TP) membranes possessed balanced WU, IEC, and conductivity values.

### Chemical Stability

**Figure 7:**
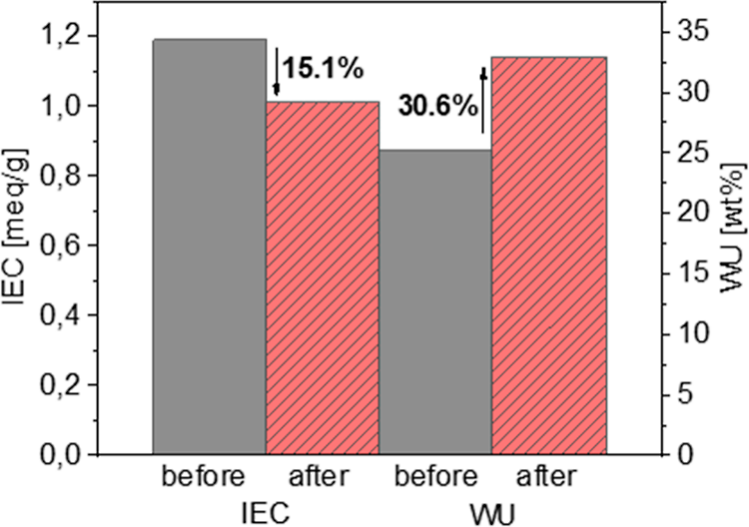
WU and IEC after Fenton’s test.

Stability toward reactive oxygen species is a crucial requirement for membranes to be used in fuel cell applications. It is well known that different types of radicals (e.g., HO^•^ and HOO^•^) form during operation, which can lead to devastating performance losses.^2,32,60^ The herein reported membranes were tested toward chemical stability via Fenton’s test (3 wt % solution of H_2_O_2_ containing 4 ppm Fe^2+^), and the IEC and WU were determined again (Figure 7).

The IEC decreased by 15.1%, and the WU increased by 30.6%. It is noteworthy that the membrane did not fully degrade within the test time, and films were retained afterward. Due to the cross-linked nature of the membrane, NMR spectroscopic analysis in solution was not possible.

**Figure 8:**
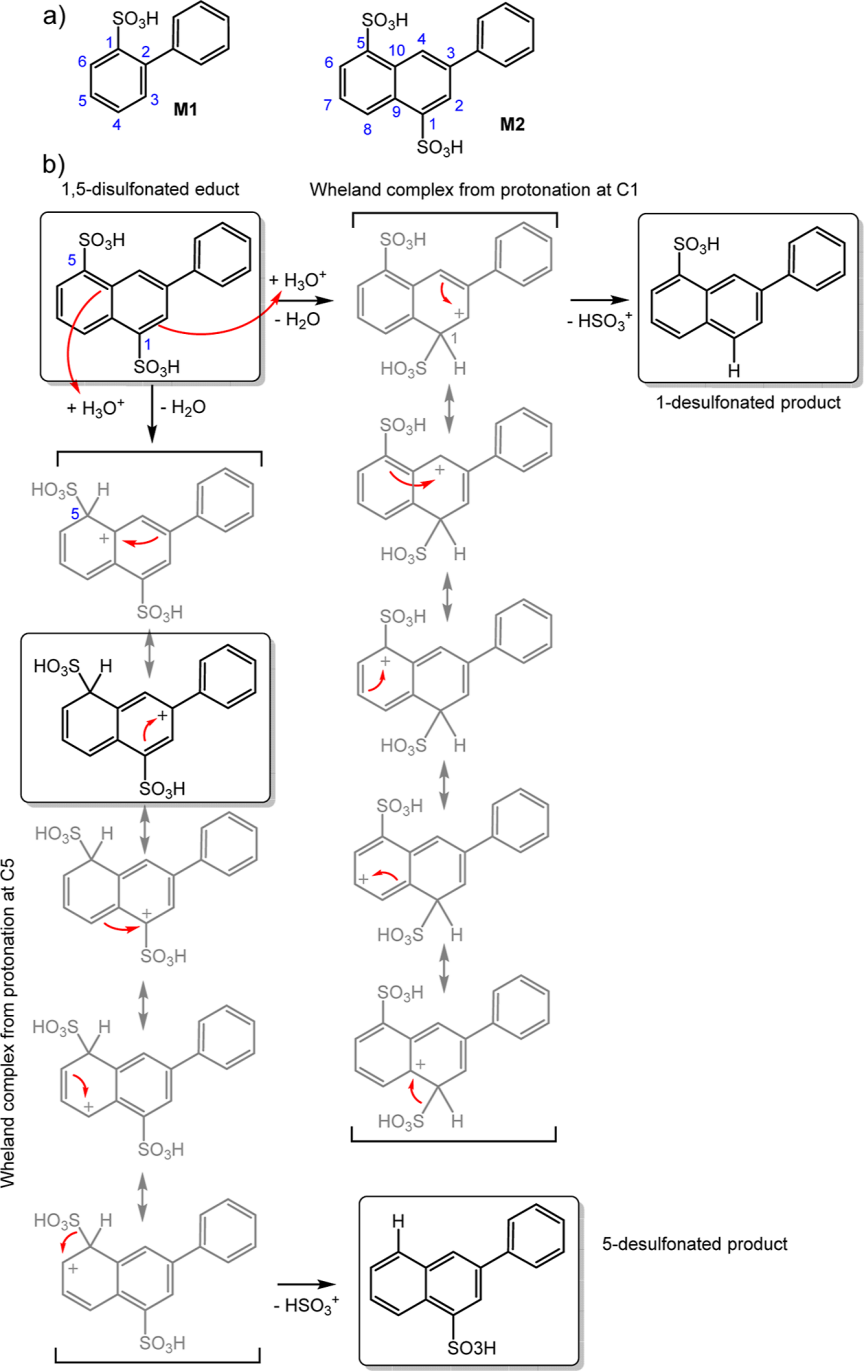
(a) Chemical structure of model compounds M1 and M2 and (b) regiochemistry effects of desulfonation reactions of M2.

The increase of WU after the application of Fenton’s conditions may be explained by a higher hydrophilicity, which in turn can arise from backbone hydroxylation.^2,32,60^ The decrease of the IEC may result from desulfonation of the AA units under acidic conditions, which is a further degradation pathway of sulfonated aromatic polymers.^18^ While clarification of mechanistic details leading to the increase in WU requires further studies, we addressed the potential desulfonation of P(AA-*alt*-*m*TP) theoretically, by carrying out DFT calculations on model compounds M1 and M2 (Figure 8a). We calculated the reaction energy for the formation of the protonated intermediates of M1 and M2 (Wheland complex), whereby for asymmetric M2, protonation in the 1- and 5-positions was considered. This energy represents a barrier for desulfonation and was used to estimate a relative stability compared to sulfonated polyphenylenes. While the protonation energy for M1 at the C1 position is 53 kJ/mol, it is 75 kJ/mol at C1 and 57 kJ/mol at C5 for M2, respectively. For details on the methodology, we refer to the Supporting Information. These values show a small increase of +4 kJ/mol and a larger increase of +22 kJ/mol for protonation of M2 when compared to M1. The discrepancy in barrier heights between protonation at 1- and 5-positions of M2 can be explained on the basis of individual mesomeric contributions to the Wheland complexes, see Figure 8b. While for both cases, for protonation at C-1 and C-5, there is an equal number of mesomeric structures and energetically disfavored contributions are present (e.g., positive charge next to the sulfonic acid group), protonation at C-5 produces a mesomeric contribution with a benzylic carbenium ion not possible for protonation at C-1. This is of course a result of the asymmetry of M2, which we expect to vanish for the symmetrically substituted naphthalene-1,5-disulfonic acid moieties in P(AA-*alt-m*TP). In other words, the phenyl ring attached to M2 stabilizes the Wheland complex for desulfonation at C-5, hence facilitates this reaction, but the resulting barrier height is still slightly larger compared to M1. Thus, from these results, model compound M2 is expected to be at least as stable against desulfonation than M1, making P(AA-*alt-m*TP) a competitive polymer for PEMs.

## Conclusions

In conclusion, we have used readily available 1,5-naphthalenedisulfonic acid (“Armstrong’s acid”) as a novel building block for the preparation of sulfonated polyphenylenes. To prepare alternating copolymers with large molar masses, we have applied and optimized Suzuki polycondensation. Usage of *m*-terphenyl instead of *m*-phenyl as the comonomer was key to achieve large molar mass and toughness of films. Furthermore, a protection/deprotection strategy based on neopentyl sulfonates was successfully implemented. These side chains improve solubility and are base stable. In contrast to deprotection in the solid state, solution-based deprotection at 150 °C eliminated isopentylene quantitatively. The large IEC arising from doubly sulfonated AA led to water-soluble copolymers after deprotection, which were cast to get membranes. Thermal cross-linking rendered the membranes water-insoluble and allowed to tune properties. By optimizing the cross-linking protocol, we were able to restrict WU to 50 wt %, to retain a high IEC of 2.33 mequiv/g, and to achieve a good proton conductivity of 85 mS/cm. The theoretical stability of AA copolymers against desulfonation under acidic conditions probed by DFT calculations indicated that the 1,5-disulfonated naphthalene building block is at least as stable as sulfonated polyphenylenes. These results demonstrate that polyphenylenes based on Armstrong’s acid are a promising avenue toward new polymeric proton conductors with improved properties, for which Suzuki cross-coupling chemistry offers ample modification of chain architecture.

## Supporting Information Available

Details of synthesis; molecular characterization; DFT; additional thermal characterizations; membrane preparation; protection/deprotection; determination of WU, IEC, and conductivity; and Fenton’s test (PDF)

## Abbreviations

AA: Armstrong’s acid
AA-NP: dineopentyl-3,7-dibromnaphthalin-1,5-disulfonate
*m*P: 1,3-bis(4,4,5,5-tetramethyl-1,3,2-dioxaborolan-2-yl)benzene
*m*TP: 3,3″-bis(4,4,5,5-tetramethyl-1,3,2-dioxaborolan-2-yl)-1,1′:3′,1″-terphenyl
P(AA-NP-*alt*-mP): poly[(1,5-naphthalene dineopentyl disulfonate)-*alt*-(1,3-benzene)]
P(AA-NP-*alt*-*m*TP): poly[(1,5-naphthalene dineopentyl disulfonate)-*alt*-(1,1′:3′,1″terphenyl)]
P(AA-*alt*-*m*TP): poly[(1,5-naphthalene disulfonic acid)-*alt*-(1,1′:3′,1″terphenyl)]
DBH: 1,3-dibromo-5,5-dimethylhydantoin
DoE: U.S. Department of Energy
SPC: Suzuki polycondensation
THF: tetrahydrofuran
DMAc: dimethylacetamide
tol: toluene
CHCl_3_: chloroform
DMF: dimethylformamide
NMR: nuclear magnetic resonance
WMR: Wagner–Meerwein rearrangement
TGA–MS: thermogravimetric analysis coupled with mass spectrometry
*m*/*z*: mass-to-charge ratio
DMSO: dimethyl sulfoxide
IR: infrared
WU: water uptake
IEC: ion-exchange capacity
ROS: reactive oxygen species
