# Supplementary material for: Proton-Conducting Membranes from Polyphenylenes Containing Armstrong’s Acid
Source: Macromolecules. 2024 Jan 30;57(3):1238–47. doi: 10.1021/acs.macromol.3c02123 (PMC10870345; doi:10.1021/acs.macromol.3c02123)
Supplement: Supplementary file 1 — ma3c02123_si_001.pdf [file ma3c02123_si_001.pdf]

Supporting information

Proton-conducting membranes from polyphenylenes containing Armstrong´s acid

*Andy Künzel-Tenner[1], Christoph Kirsch[2], Oleksandr Dolynchuk[3], Leonard Rößner[4], Maxime Wach[5], Fabian Kempe[1,$], Thomas von Unwerth[5], Albena Lederer[6,7], Daniel Sebastiani[2], Marc Armbrüster[4], Michael Sommer[1,8]**

[1]Institut für Chemie, Polymerchemie, Technische Universität Chemnitz, Straße der Nationen 62, 09111 Chemnitz, Germany

[2]Institut für Chemie, Theoretische Chemie, Martin-Luther-Universität Halle-Wittenberg, Von-Danckelmann-Platz 4, 06120 Halle, Germany

[3]Experimental Polymer Physics, Martin Luther University Halle-Wittenberg, Von-Danckelmann-Platz 3, 06120 Halle, Germany

[4]Institut für Chemie, Materialien für innovative Energiekonzepte, Technische Universität Chemnitz, Straße der Nationen 62, 09111 Chemnitz, Germany

[5]Institut für Automobilforschung, Technische Universität Chemnitz, Reichenhainer Straße 70, 09126 Chemnitz, Germany

[6]Leibniz Institut für Polymerforschung Dresden e. V., Hohe Straße 6, 01069 Dresden, Germany

[7]Department of Chemistry and Polymer Science, Stellenbosch University, Private Bag X1, Matieland 7602, South Africa

[8]Forschungszentrum MAIN, TU Chemnitz, Rosenbergstraße 6, 09126 Chemnitz, Germany

[$]Present address: Forschungszentrum Jülich GmbH, Helmholtz-Institute Münster, IEK-12, Corrensstr. 46, 48149 Münster, Germany

Table of contents

1. Chemicals and Methods 3
1.1. Chemicals 3
1.2 Methods 3
2. Synthesis 10
2.1. Synthesis of dipyridinium-3,7-dibromonaphthalene-1,5-disulfonate (2) 10
2.2 Synthesis of 3,7-dibromonaphthalene-1,5-disulfonic acid dichloride (3) 11
2.3 3,7-Dibromonaphthalene-1,5-neopentyldisulfonate (AA-NP 4) 12
2.4 General procedure for polymerization (exemplified by entry P8) 13
3. Characterization 15

1. Chemicals and Methods

1.1. Chemicals

1,3-Bis(4,4,5,5-tetramethyl-1,3,2-dioxaborolan-2-yl)benzene ("*m*P", 97 %, BLD Pharm, China), 3,3''-dibromo-1,1':3',1''-terphenyl (99 %, BLD Pharm, China), 1,5-napthalenedisulfonic acid pentahydrate (Sigma-Aldrich, Germany), 1,3-dibromo-5,5-dimethylhydantoin ("DBA", abcr, Germany), pyridine (Grüssing, Germany), chlorosulfonic acid (ThermoFisher scientific, Germany), neopentyl alcohol (Alfa Aesar, USA), potassium acetate (technical grade), 1,1′-bis-(di-tert.-butylphosphino-)ferrocene-palladium dichloride (BLD Pharm, China), potassium phosphate (stored under inert conditions, abcr, Germany), toluene (Grüssing, Germany), dimethylacetamide (HPLC grade, Fisher scientific, USA), 1M hydrochloric acid (Grüssing, Germany) and hexane (technical grade) were used as received. Dimethylacetamide was stored over molecular sieve (4Å) and under argon atmosphere after degassing with argon. Toluene was distilled prior to use and stored over molecular sieve (4 Å) and under argon atmosphere. 3,3''- Bis(4,4,5,5-tetramethyl-1,3,2-dioxaborolan-2-yl)-1,1':3',1''-terphenyl ("***m*TP**") was synthesized according to a previously reported procedure.[1]

1.2 Methods

Solution NMR spectroscopy. NMR spectra were recorded on a Bruker Avance NEO 600 spectrometer (^{1}H: 600 MHz, ^{13}C: 125 MHz). The spectra were referenced to the residual solvent peak ($CDCl_3$: $\delta(^{1}H)$ = 7.26 ppm, $\delta(^{13}C)$ = 77.0 ppm, DMSO: $\delta(^{1}H)$ = 2.50 ppm, $\delta(^{13}C)$ = 39.51 ppm).

Solid state NMR spectroscopy. Solid-state nuclear magnetic resonance spectroscopy (S-NMR) was performed at 9.4 T on a Bruker Avance 400 spectrometer equipped with double-tuned probes capable of MAS (magic angle spinning). The finely powdered samples were packed into

3.2 mm rotors (OD) made of zirconium oxide spinning at 15 kHz. $^{13}C-\{^{1}H\}$-CP-MAS NMR spectra were acquired using cross polarization (CP) technique with contact time of 3 ms to enhance sensitivity, a recycle delay of 1.5 s, and ^{1}H decoupling during acquisition using a TPPM (two pulse phase modulation) puls sequence. The spectra are referenced with respect to tetramethyl silane (TMS) using TTSS (tetrakis(trimethylsilyl)silane) as a secondary standard (3.55 ppm for ^{13}C, 0.27 ppm for ^{1}H). All spectra were acquired at room temperature (25 °C).

Size exclusion chromatography. SEC measurements of all samples were carried out on PolarGel-M columns (300 x 7.5 mm from Agilent Technologies, US), connected in series with a HPLC-Pump 1200(Agilent Technologies, US), a KNAUER K2301 RI detector (Knauer GmbH, DE), and a MiniDAWN-LS detector "TREOS II" (Wyatt Technology) at 25°C. N,N-Dimethylacetamide containing 3 g/L LiCl was used as eluent at a flow rate of 1.0 mL/min. The absolute molar masses and molar mass distributions were calculated using Astra 7.3.2 software (Wyatt Technology, US). The refractive increment values dn/dc for calculation of the molar mass were calculated using the same software and assuming full separation of the injected samples or using external determination if required.

Differential scanning calorimetry. DSC measurements were carried out on a DSC 2500 (TA Instruments) under nitrogen atmosphere. Heating and cooling rates were 10 K/min. The mass of the samples for each measurement was approx. 5 mg.

Thermogravimetric analysis. TGA measurements were done on a TGA/DSC3+ (Mettler-Toledo) within the temperature range 30 °C to 650 °C at a heating rate of 10 K/min under argon.

Small- and wide-angle X-ray scattering (SAXS and WAXS). WAXS and SAXS experiments were performed in a SAXSLAB laboratory (Retro-F) equipped with an AXO microfocus X-ray source and an AXO multilayer X-ray optics (ASTIX) as a monochromator for Cu-Kα radiation (λ = 0.15418 nm). A PILATUS3 R 300K detector from DECTRIS was used to record the 2D

scattering patterns. The measurements were performed in transmission geometry under vacuum at room temperature; the sample to detector distance was approximately 85 mm for WAXS and 1035 mm for SAXS. The isotropic SAXS and WAXS patterns were integrated over the azimuthal angle to generate the 1D curves of *I(q)*.

Thermogravimetric analysis coupled to mass spectrometry

TG/MS (Netzsch STA 449 F3 Jupiter, Pfeiffer Omnistar) measurements were conducted in Al_2O_3 crucibles using a heating rate of 3 K/min. During the measurements, helium (Air Liquide, 99.999%) was flushed through the system at 40 mL/min using mass flow controllers (Bronkhorst EL-FLOW). Background correction was conducted by subtraction of blank measurements under identical conditions. The ion current for m/z = 70 and m/z = 81 signals was used as an indicator for isopentylene and sulphurous acid, respectively.

MALDI-TOF MS. Matrix-assisted laser desorption/ionization mass spectra were taken using BRUKER autoflex MALDI-TOF instrument in negative ion and reflector operating modes. The laser of this instrument is a smartbeam-II with a wavelength of 355 nm. The software for measuring and evaluating the spectra is flexControl 3.4 and flexAnalysis 3.4.

Samples were prepared on a standard sample plate (Bruker "MTP 384 target plate ground steel BC"). Sample spot preparation was as follows. The Sample (1.0 mg/mL a suitable solvent) was hand-spotted onto a MALDI sample plate and air-dried. Afterwards the substance was spotted on top of the sample spot as a matrix and air dried before analysis.

Density functional theory calculations. DFT calculations[2,3] were performed at the B3LYP level[4–7] using the ORCA program package[8,9], version 5.0.3, with def2-TZVP basis sets[10], a D3 dispersion correction with Becke-Johnson damping[11,12] and TightSCF convergence criteria. To account for solvent effects, the conductor-like polarizable continuum model (CPCM)[13] was applied for implicit water solvation. After geometry optimizations of the two model compounds

M1 and **M2**, their respective Wheland complexes, H_2O and H_3O^+ were carried out applying a BFGS optimizer and NormalOpt convergence criteria, reaction energies for the protonation of **M1** and **M2** were obtained as the differences in energy of the relaxed product (Wheland complex of **M1**/**M2** + H_2O) and reactant (**M1**/**M2** + H_3O^+) structures.

Cross-linking under inert atmosphere

The membranes were cut into 1x1 cm^2 pieces, placed in quartz-glass vials and flushed with argon three times. The vials were sealed and placed into an oven, following a thermal protocol from *Di Vona et al.*.[14] After cross-linking, the vials were opened and the membranes were taken out cautiously.

Water uptake

To determine water uptake (WU), cast membranes P(AA-*alt*-*m*TP) from DMSO were dried at 60 °C for 24 h under vacuum, weighted and immersed in deionized water at 80 °C for 24 h. The membranes were carefully dried and weighted, and WU was calculated using the following equation

$$WU(wt\%)=\frac{(w_{soaked}-w_{dry})}{w_{dry}} \quad (2)$$

with w_{soaked} representing the weight of the immersed membrane and w_{dry} being the weight of the dried membrane.

Ion exchange capacity (IEC)

The ion exchange capacity was determined via titration. Therefore, the air-dried, acidified membrane (1M HCl, 24 h) was set in a brine solution for 24 h. The membrane was taken out of the vessel and the solution was back titrated with 0.05 M NaOH against cyanidin. With the obtained data, IEC was calculated using the following equation:

$$IEC=\frac{V(NaOH)*c(NaOH)}{m(Membrane)_{dry}} \quad (3)$$

where V(NaOH) is the amount of NaOH consumed during the titration.

Mechanical properties

Tensile Testing were carried out on a Linkham TST-350. Prior to testing, the samples were prepared with a standard shape (DIN 53504 type 3, equal to ISO 37 type 4), with a thickness between 100 and 150 μm. The observed stress is defined as the engineering stress, which is calculated based on the initial cross-sectional area of the specimen at its midpoint when it experiences 0 % strain. It's important to note that the actual or true stress just before fracture is anticipated to be significantly greater. This is attributed to the ongoing thinning of the specimen as it undergoes stretching. The presented strain is defined as engineering strain, which quantifies the percentage elongation concerning the initial gauge length of the specimen, set at 15 mm. The strain rate applied during the test was 2 mm/min.

Proton conductivity measurements

Proton conductivity was measured via a modified through-plane setup reported in the literature.[15] Instead of platinum, 5x1 cm^2 gold plates were used as electrodes. The membranes were cut into 1x1 cm^2 films and cross-linked, if needed, immersed in 1M HCl for at least 24 h, taken out and rinsed with deionized water carefully. The thickness of the membranes was measured 10 times with a layer thickness measurement device from List-Magnetik and the calculated mean value was used for further calculations. Then, the membrane was fixed in the proton conductivity setup and the whole was placed into a humidity chamber from Binder. Proton conductivity was measured at 90 % RH at 80 °C, using electrochemical impedance spectroscopy (EIS) over a frequency range from 100 Hz to 7 MHz and a voltage amplitude of 10 mV. The resistance was read out at the high frequency intercept of the obtained curve in the Nyquist plot with the x-axis and the measurement was stopped once this value converged. The proton conductivity was calculated based on the following equation:

$$\sigma = \frac{l}{R \times A} \quad (1)$$

Where σ is the proton conductivity (S/cm), l is the thickness of the membrane (cm), A is the area of the membrane (cm^2) and R is the obtained resistance (Ω).

Fenton's Test

Cross-linked membranes, were immersed in a 3 wt.-% solution of H_2O_2 containing 4 ppm Fe^{2+} (with the source of Fe^{2+} being $FeSO_4$ * 7 H_2O). The solution was then heated up to 80 °C for 1 h. Afterwards, the membranes were carefully rinsed with deionized water and dried with a filter paper before further analysis.

2. *Synthesis*

2.1. *Synthesis of dipyridinium-3,7-dibromonaphthalene-1,5-disulfonate (2)*

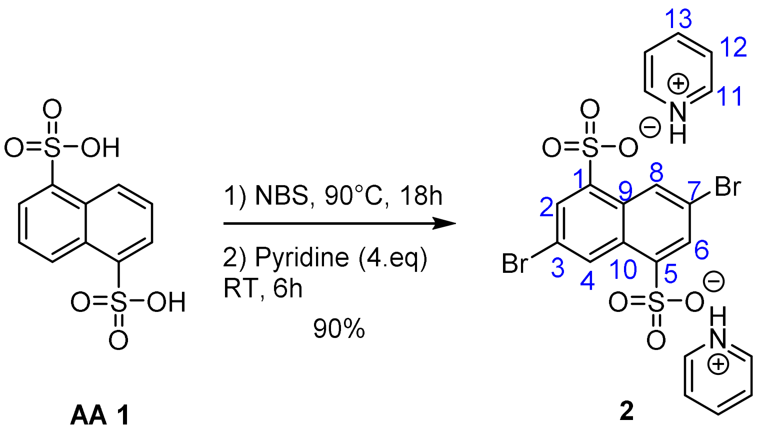

AA 1 **2**

Compound **2** was synthesized via an adopted protocol reported previously.[16] The temperature was kept at 90 °C during the reaction. The resulting solid was dissolved in ethanol (200 mL). To this solution, pyridine (100 mL) was added and the solution was allowed to stir for 6 h. Product **2** was obtained yellow powder, which was isolated by filtration and dried in a vacuum oven over night at 30 °C (113.12 g, 187.2 mmol, 90 %).

^{1}H NMR (DMSO-d$_6$, 600 MHz, 25 °C): δ 9.04 ppm (d, $^{5}J_{H\text{-}2,H\text{-}1}$ = 0.8 Hz, 2 H, *H-2 and H-6*), 8.85 ppm (d, $^{3}J_{H\text{-}3,H\text{-}4}$ = 5 Hz, 4H, *H-11*), 8.41 ppm (t, $^{3}J_{H\text{-}5,H\text{-}4}$ = 7.8 Hz, 2H, *H-13*), 8.02 ppm (d, $^{5}J_{H\text{-}1,H\text{-}2}$ = 0.8 Hz, 2 H, *H-4 and H-8*), 7.91 ppm (t, $^{3}J_{H\text{-}4,H\text{-}5}$ = 6 Hz, $^{3}J_{H\text{-}4,H\text{-}3}$ = 7 Hz, 4H, *H-12*).

^{13}C NMR (DMSO-d$_6$, 125 MHz, 25 °C): δ 146.25 ppm (s, 2 C, *C-13*), 145.22 ppm (s, 2C, *C-1 and C-5*), 142.29 ppm (s, 4H, *C-11*), 130.57 ppm (s, 2C, *C-2 and C-6*), 129.30 ppm (s, 2C, *C-3 and C-7*), 127.87 ppm (s, 2C, *C-4 and C-8*), 127.16 ppm (s, 4C, *C-12*), 118.46 ppm (s, 2C, *C-9 and C-10*).

MS (MALDI/TOF) m/z: [M] Calcd. for $C_{10}H_4Br_2O_6S_2^{2-}$: 221.89 (100%), 220.89 (51.4%), 222.89 (48.6%); Found 221.81 (100%), 220.81 (51.4%), 222.82 (48.6%).

2.2 Synthesis of 3,7-dibromonaphthalene-1,5-disulfonic acid dichloride (3)

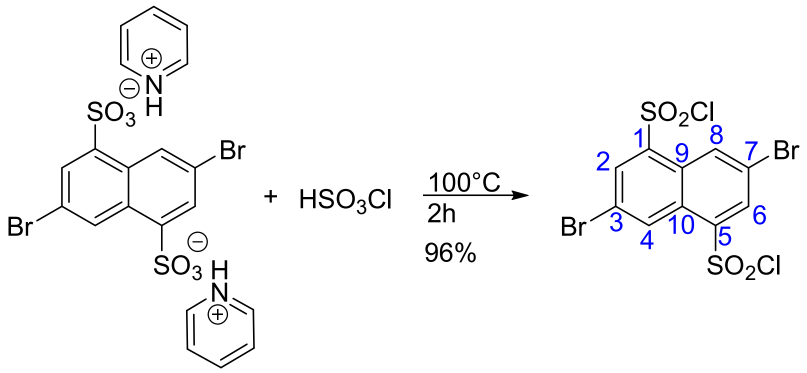

2 **3**

The synthesis was adapted from a previously reported procedure.[17]

In a 500 mL round bottom flask, **2** (41.88 g, 69.3 mmol) was added to chlorosulfonic acid (100 mL, large excess) and the whole was heated to 100 °C. After 2 hours, the resulting dispersion was added dropwise onto ice water. The resulting solid was filtered, washed with cold water and cold ethanol afterwards and dried under reduced pressure for 12 h at 40 °C. (32.13 g, 66.5 mmol, 96 %).

^{1}H NMR ($CDCl_3$, 600 MHz, 25 °C): δ 9.31 ppm (s, 2H, *H-2 and C-6*), 8.64 ppm (s, 2H, *H-4 and H-8*).

^{13}C NMR ($CDCl_3$, 125 MHz, 25 °C): δ 140.5 ppm (s, 2C, *C-1 and C-5*), 134.55 ppm (s, 2C, *C-2 and C-6*), 134.16 ppm (s, 2C, *C-1 and C-5*), 128.05 ppm (s, 2C, *C-9 and C-10*), 122.14 ppm (s, 2C, *C-3 and C-7*).

MS (MALDI/TOF) m/z: [M] Calcd. for $C_{10}H_4Br_2Cl_2O_4S_2$: 481.73 (100%), 483.72 (63.9%), 479.73 (51.4%); Found: 481.69 (100%), 483.71 (63.9%), 479.64 (51.4%).

2.3 3,7-Dibromonaphthalene-1,5-neopentyldisulfonate (AA-NP 4)

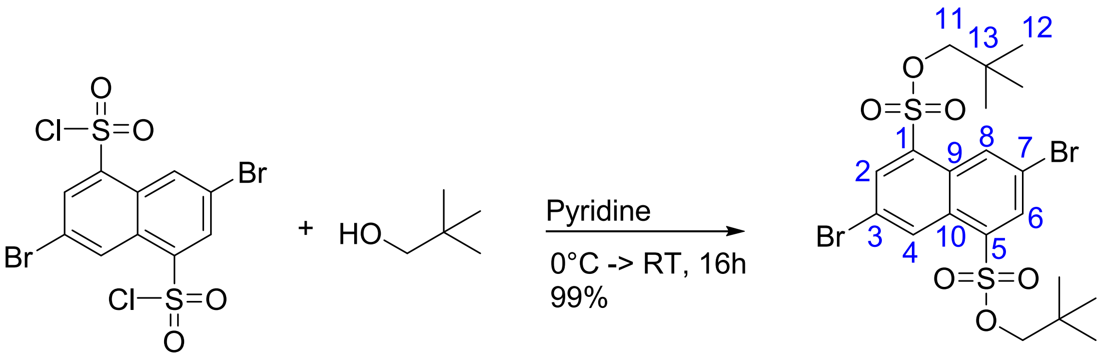

3 **AA-NP 4**

In a 3000 mL flask, to a solution of neopentylalcohol (14.55 g, 165.1 mmol) in pyridine (100 mL), 3,7-dibromonaphthalene-1,5-disulfonicacid dichloride (20 g, 41.4 mmol) was added in portions at 0 °C. After completion of the addition, the solution was kept at 0 °C for 2 h, allowed to warm to room temperature and stirred for another 14 h. The solids were filtered, washed with ethanol (150 mL) and recrystallized from a THF/acetone mixture (2:1, 200 mL). The solids were again filtered and dissolved in chloroform, which was removed afterwards under reduced pressure to remove residual acetone. This process was repeated thrice to yield pure **AA-NP 4** as colorless powder (24.04 g, 41.0 mmol, 98%).

^{1}H NMR ($CDCl_3$, 600 MHz, 25 °C): δ 9.31 ppm (s, 2H, *H-2 and H-6*), 8.64 ppm (s, 2H, *H-4 and H-8*), 3.72 ppm (s, 4H, *H-11*), 0.89 ppm (s, 18H, *H-12*).

^{13}C NMR ($CDCl_3$, 125 MHz, 25 °C): δ 140.68 ppm (s, 2C, *C-1 and C-5*), 134.75 ppm (s, 2C, *C-2 and C-6*), 134.31 ppm (s, 2C, *C-4 and C-8*), 128.24 ppm (s, 2C, *C-9 and C-10*), 122.35 ppm (s, 2C, *C-3 and C-7*), 81.3 ppm (s, 2C, *C-11*), 32.1 ppm (s, 2C, *C-13*), 26.5 ppm (s, 6C, *C-12*).

Elemental analysis: Calcd.: C: 40.79%, H: 4.47%, S: 10.94%, found: C: 41.00%, H: 4.45 %, S: 10.80%.

2.4 General procedure for polymerization (exemplified by entry P8)

In a 12 mL screw-cap vial with a septum, **AA-NP** (100 mg, 0.17 mmol, 1.00 eq.), ***m*TP** (85.94 mg, 0.178 mmol, 1.045 eq.), potassium phosphate (218.31 mg, 1.02 mmol, 6 eq.) and Pd(dtbpf)Cl_2 (2 mol-%) were degassed under a stream of argon for 30 min. 3.5 mL dry N,N-dimethylacetamide, 3.5 mL dry toluene and 1 mL deionized and degassed water were added. The screw-cap with septum was replaced, the vial was closed and sealed with teflon tape. The mixture was stirred for 3 days at 90 °C. The reaction mixture was quenched with water, the resulting solid was filtered, washed with water, dissolved in N,N-dimethylacetamide and reprecipitated into hexanes.

P(AA-*alt*-*m*P) (Entries P1-5):

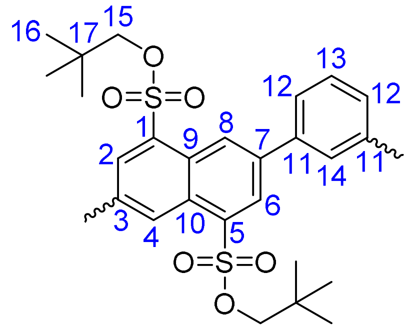

^{1}H NMR ($CDCl_3$, 600 MHz, 25 °C): δ 9.40 ppm (s, 2H, *H-4 and H-8*), 8.92 ppm (s, 2H, *H-2 and H-6*), 8.37-7.78 ppm (multiple s, 4H, *H-12, H-13 and H-14)*, 3.85 ppm (s, 4H, *H-15*) 0.8 ppm (s, 18H, *H-16*).

P(AA-NP-*alt-m*TP) (Entries P6-11):

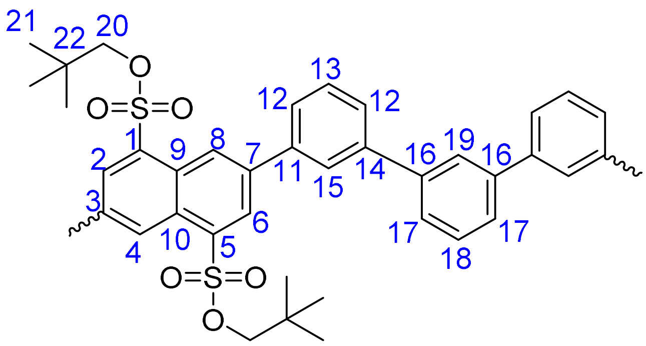

^{1}H NMR (DMSO-d_6, 600 MHz, 25 °C): δ 9.54 ppm (s, 2H, *H-2 and H-6*), 9.14 ppm (s, 2H, *H-4 and H-8*), 8.65-7.14 ppm (m, 14H, *H-12 – H-19)*, 3.62 ppm (s, 2H, *H-20*), 0.71-0.5 ppm (s, 18H, *H-21*).

P(AA-*alt-m*TP):

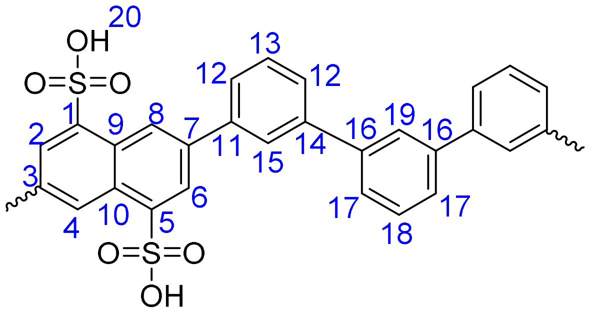

^{1}H NMR (DMSO-d_6, 600 MHz, 25 °C): δ 9.31 ppm (s, 2H, *H-2 and H-6*), 8.43 ppm (s, 2H, *H-4 and H-8*), 8.16-7.63 ppm (m, 14H, *H-12 – H-19)*, 6.01 ppm (broad s, 2H, *H-20*).

3. Characterization

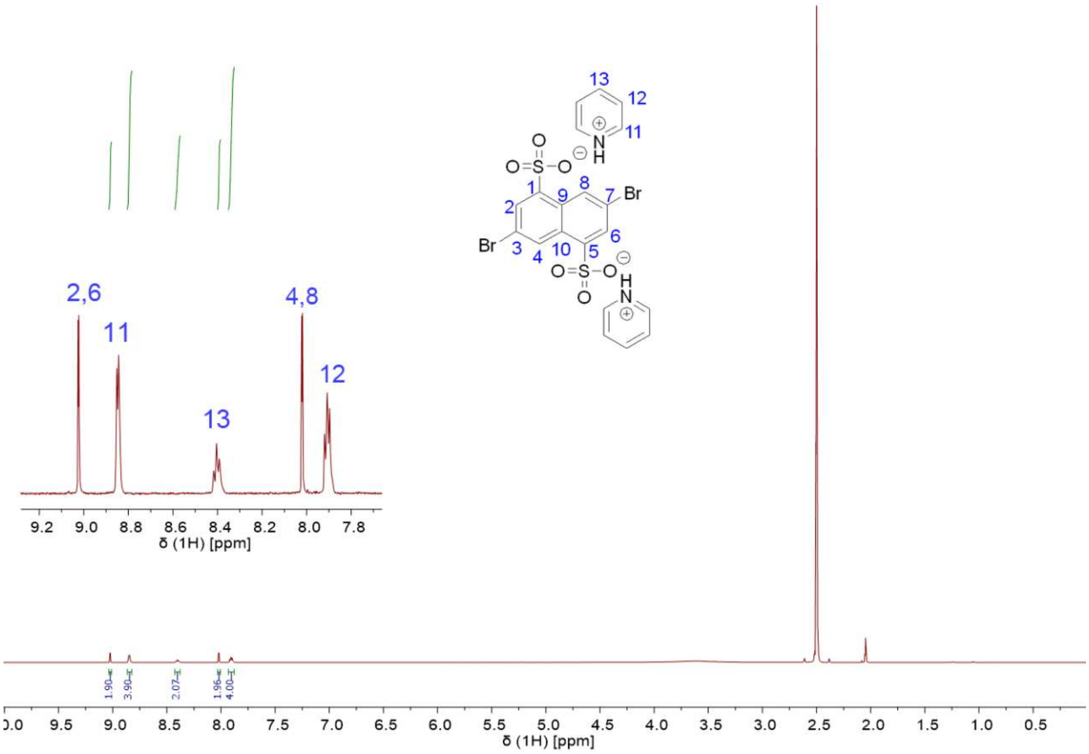

Figure S1. ^{1}H NMR spectra of compound **2** in DMSO-d_6.

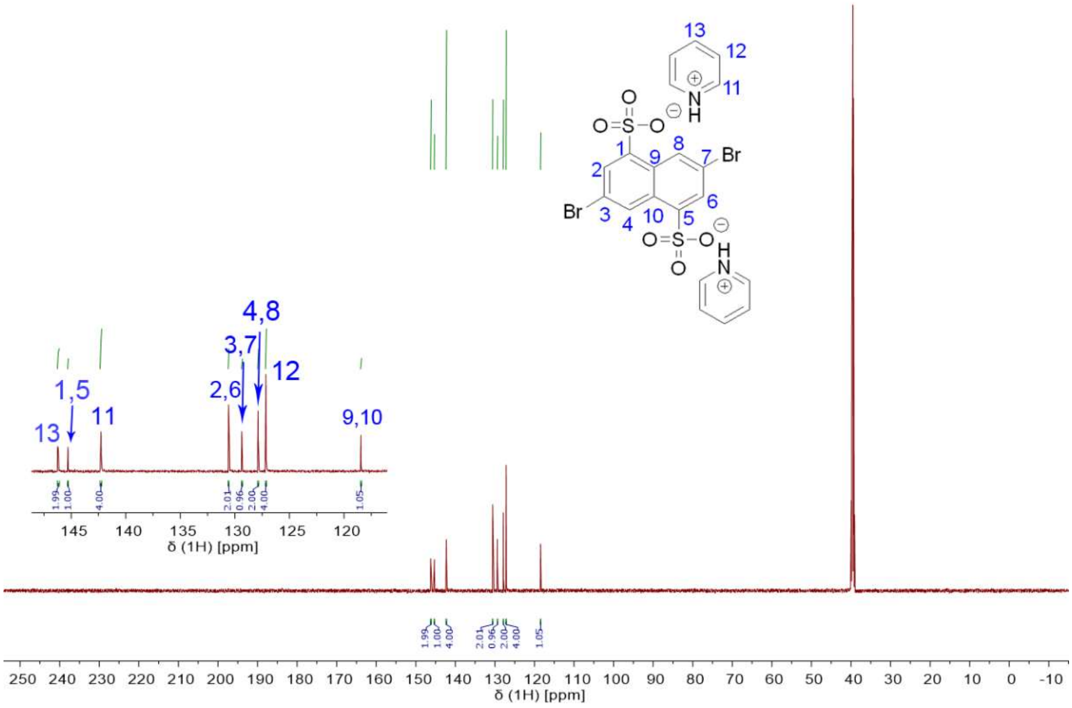

Figure S2. ^{13}C NMR spectra of compound **2** in DMSO-d_6.

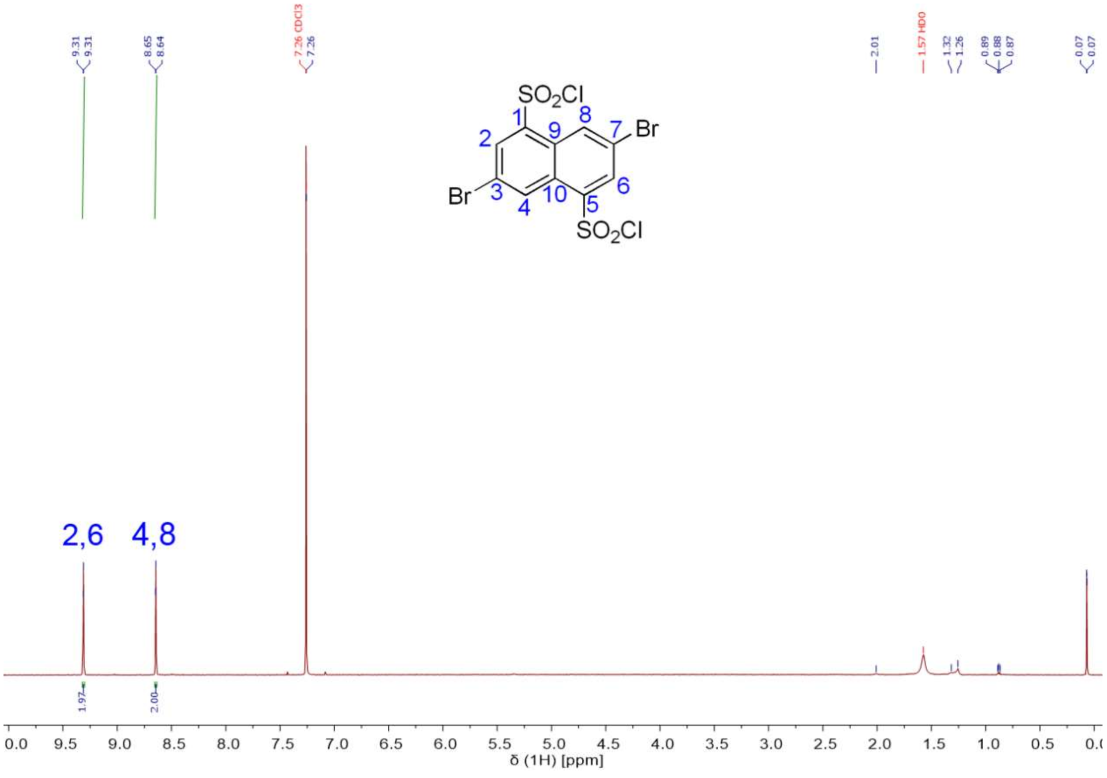

Figure S3. ^{1}H NMR spectra of compound **3** in $CDCl_3$.

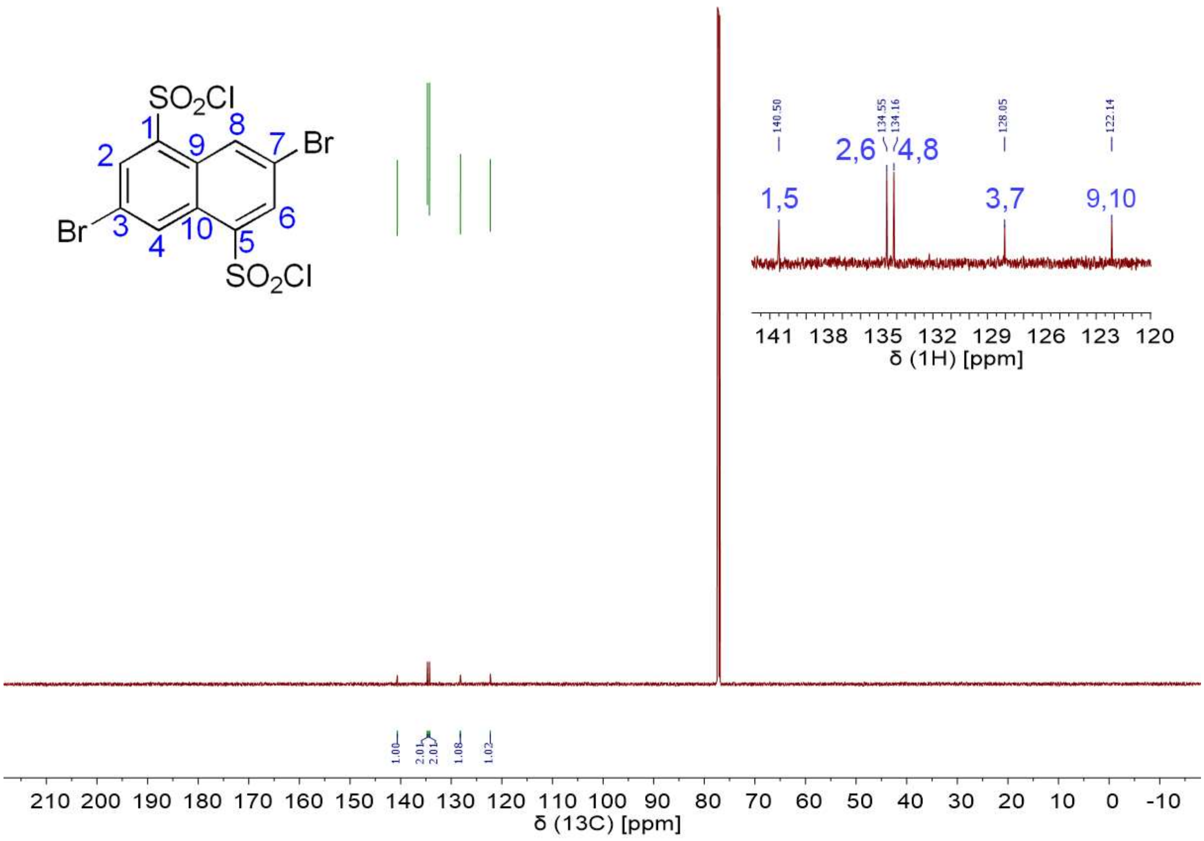

Figure S4. ^{13}C NMR spectra of compound **3** in $CDCl_3$.

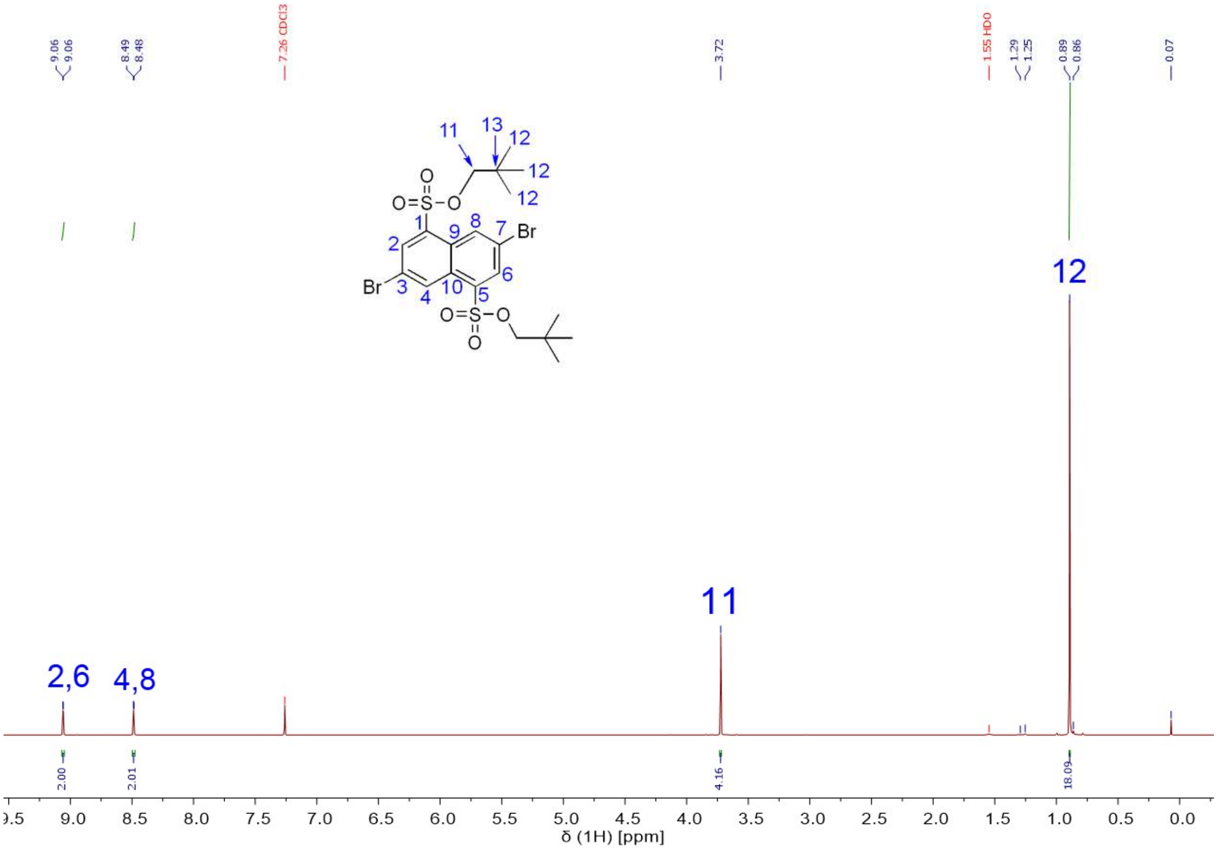

Figure S5. ^{1}H NMR spectra of compound **AA-NPA 4** in $CDCl_3$.

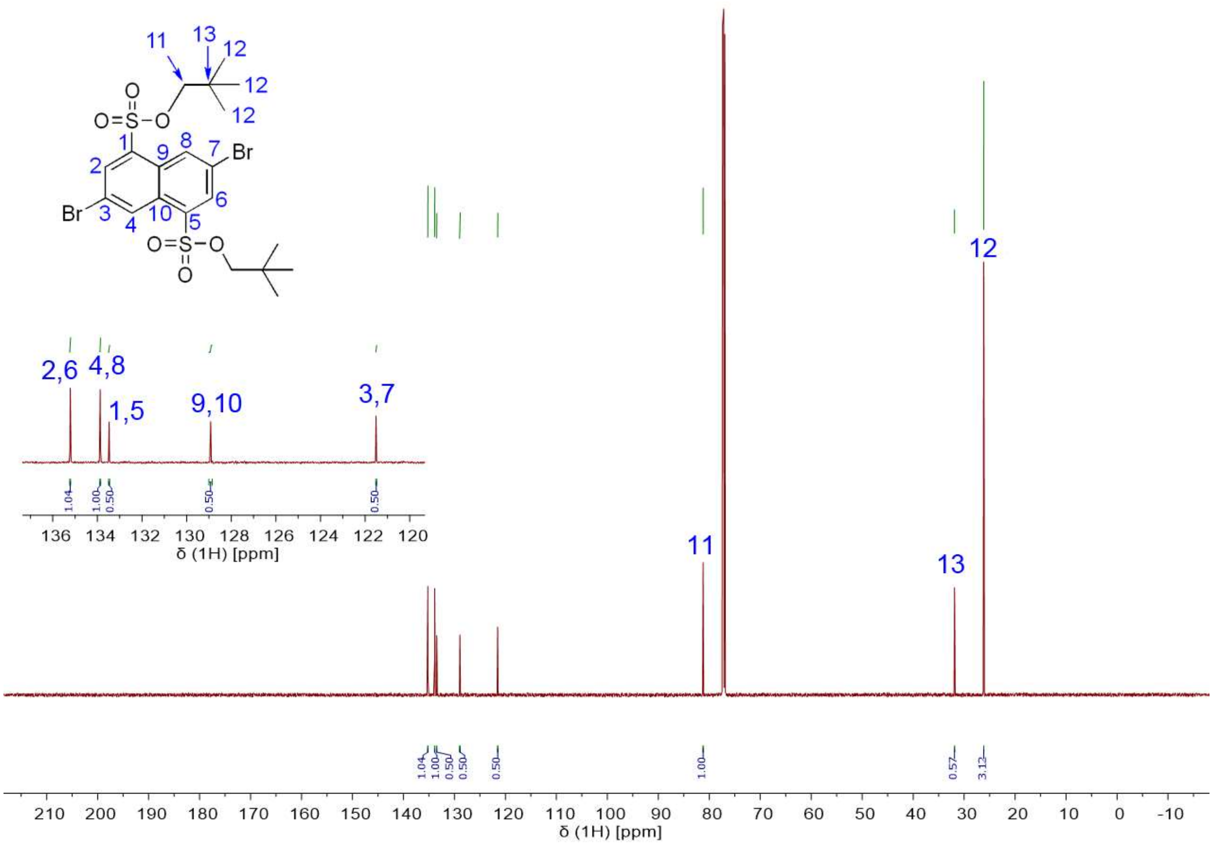

Figure S6. ^{13}C NMR spectra of compound **AA-NPA 4** in $CDCl_3$.

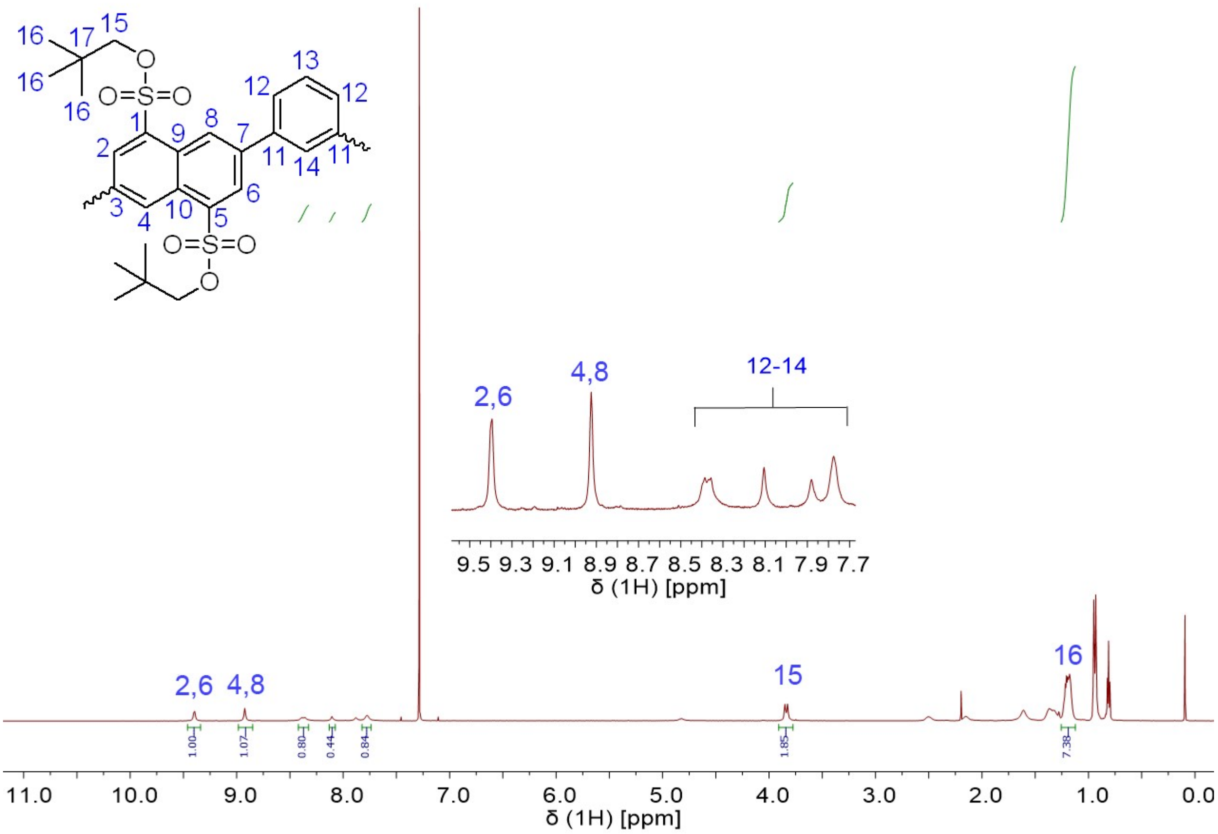

Figure S7. ^{1}H NMR spectra of polymer (**P1**) in $CDCl_3$.

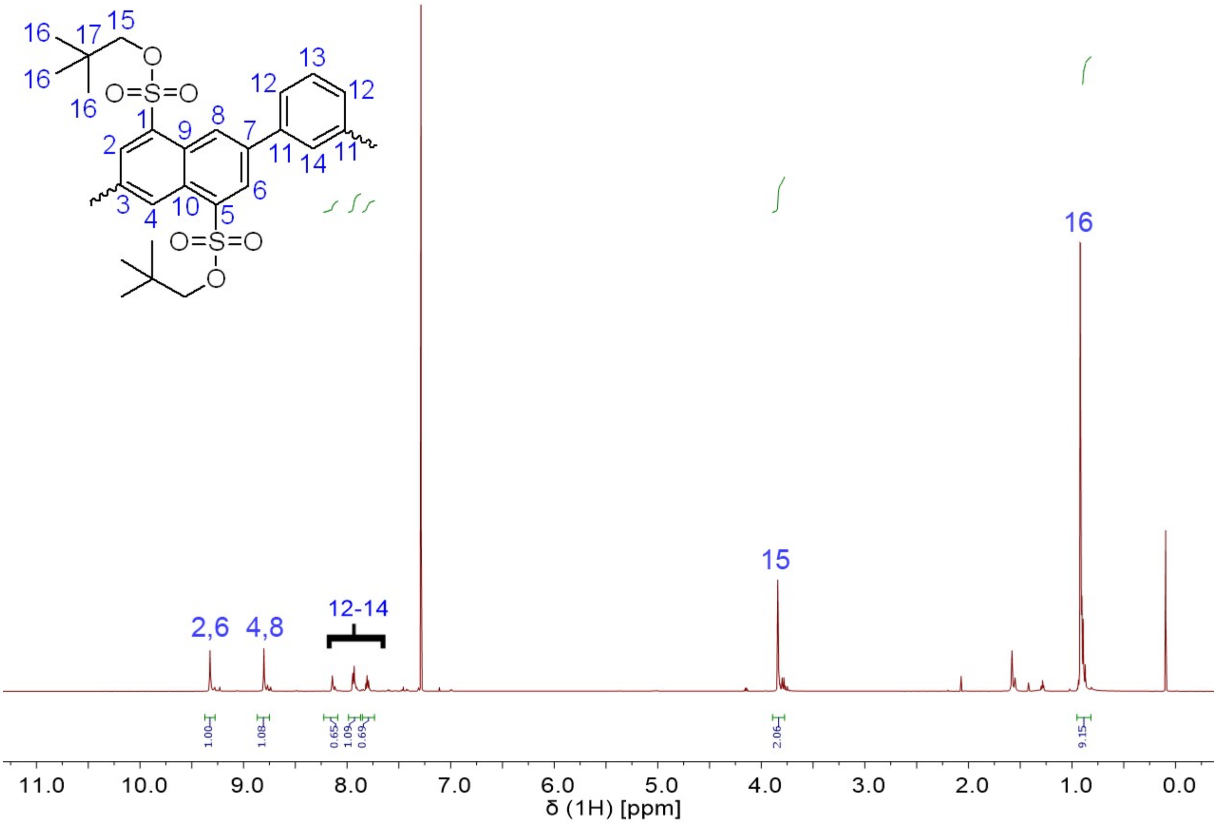

Figure S8. ^{1}H NMR spectra of polymer (**P2**) in $CDCl_3$.

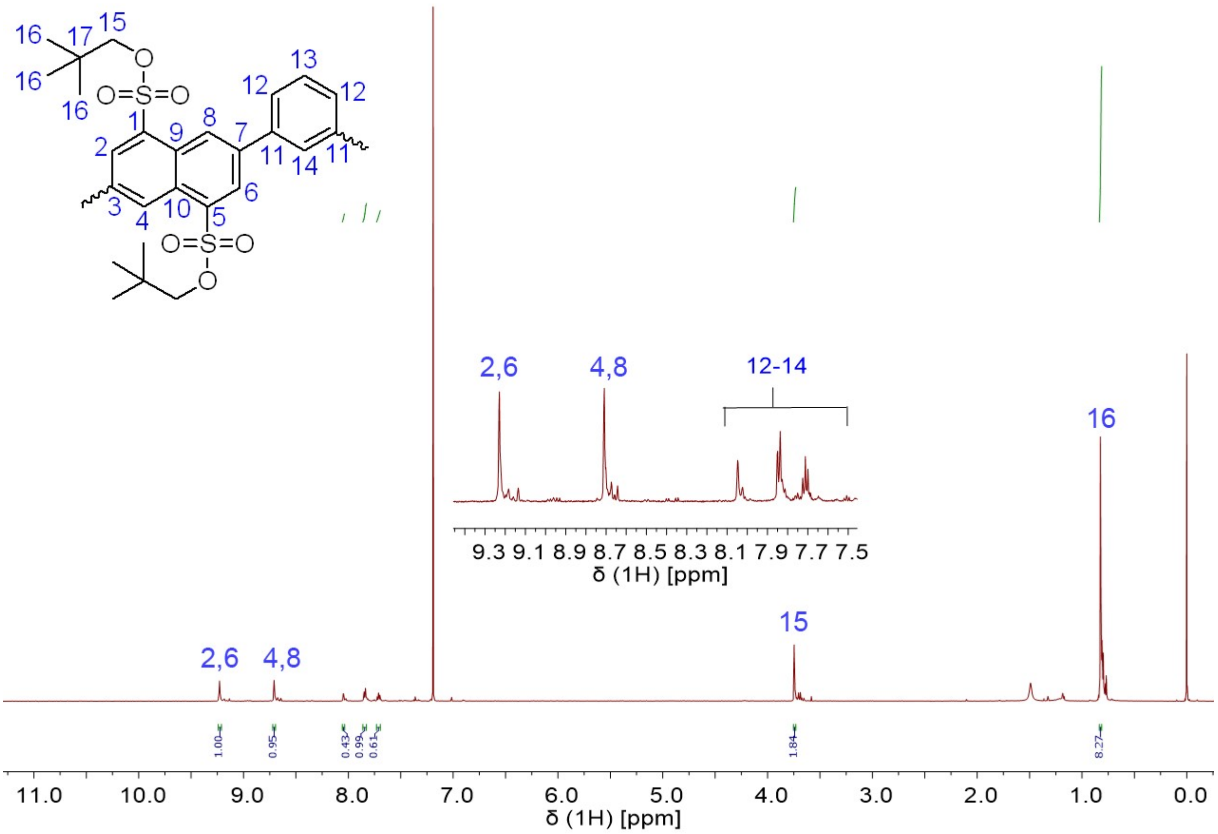

Figure S9. ^{1}H NMR spectra of polymer (**P3**) in $CDCl_3$.

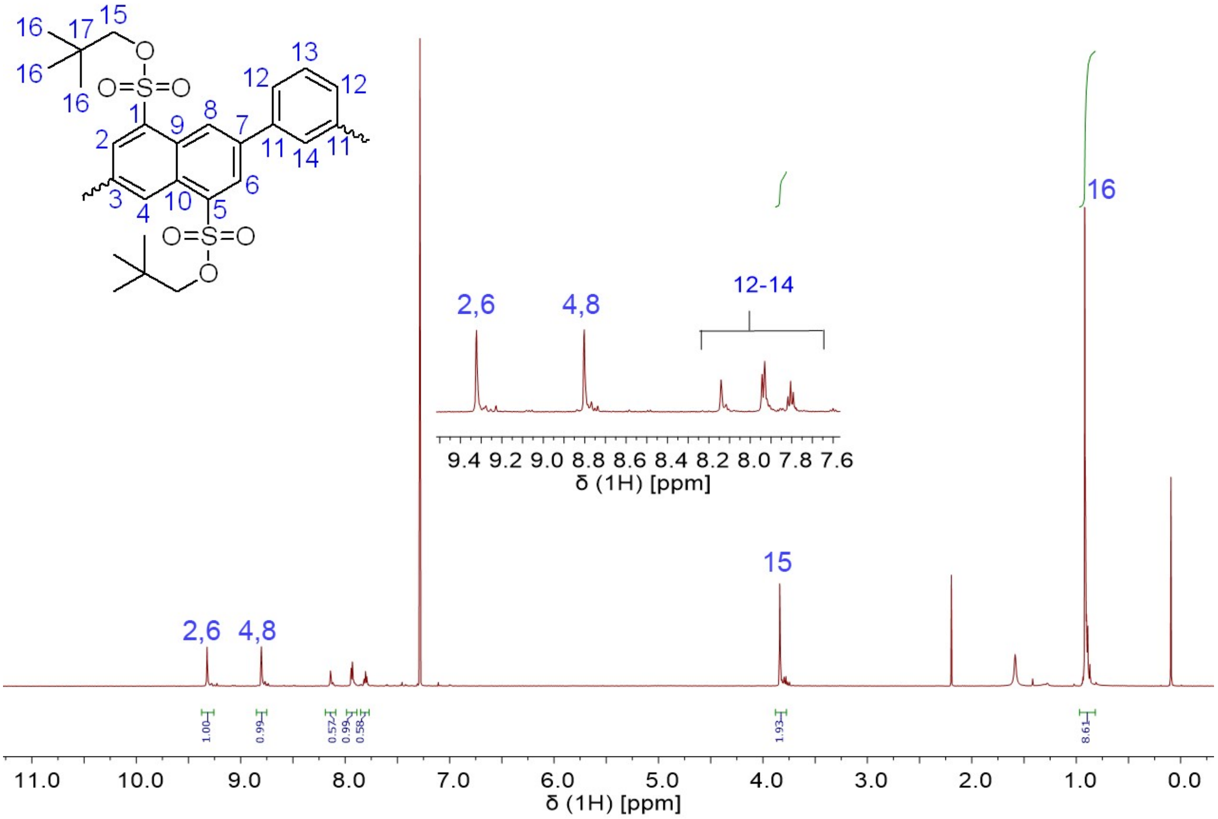

Figure S10. ^{1}H NMR spectra of polymer (**P4**) in $CDCl_3$.

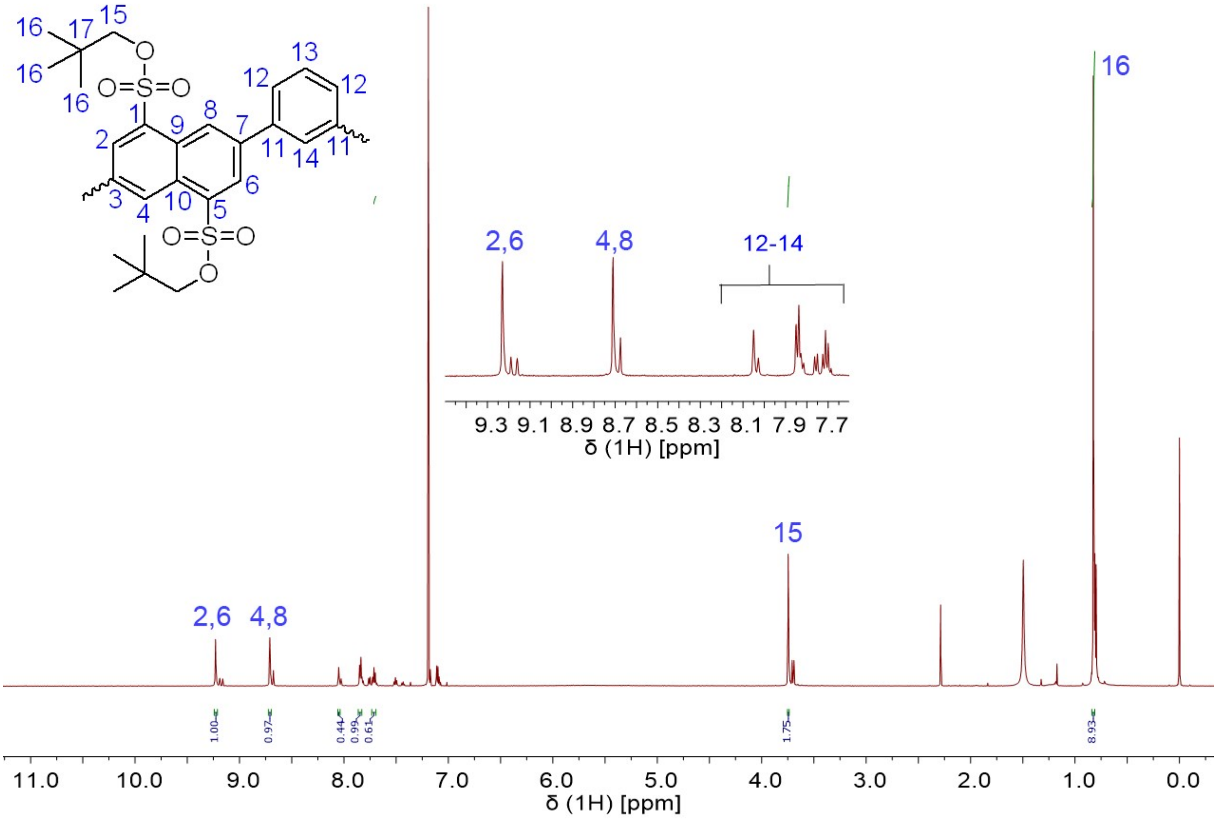

Figure S11. ^{1}H NMR spectra of polymer (**P5**) in $CDCl_3$.

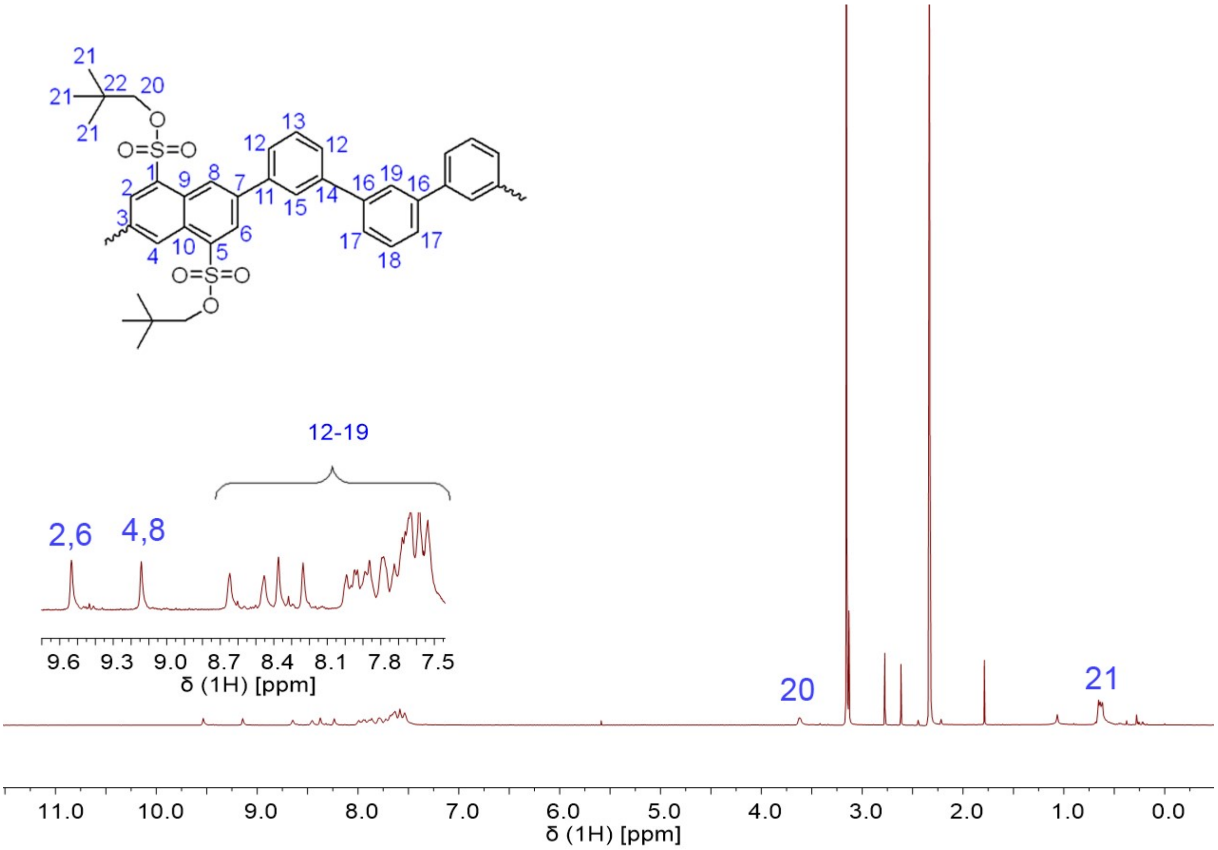

Figure S12. ^{1}H NMR spectra of polymer (**P6**) in DMSO-d_6.

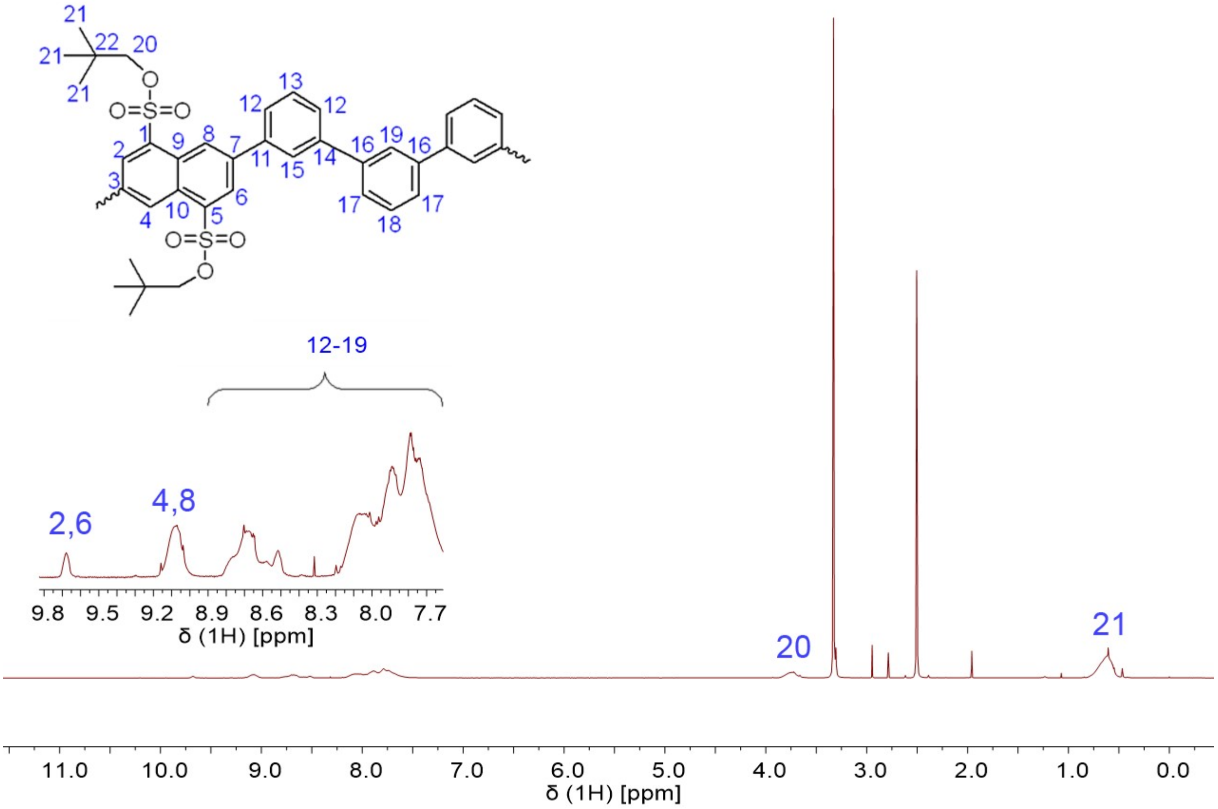

Figure S13. ^{1}H NMR spectra of polymer (**P7**) in DMSO-d_6.

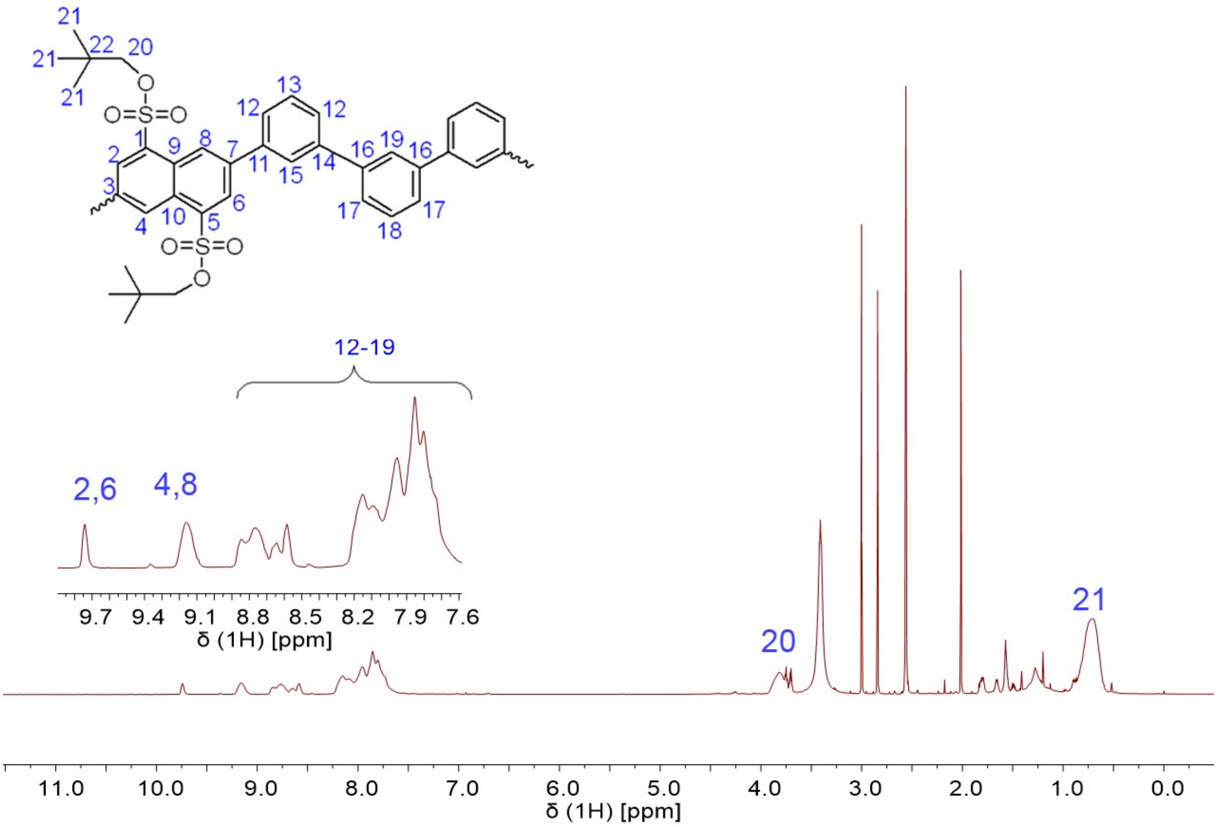

Figure S14. ^{1}H NMR spectra of polymer (**P8**) in DMSO-d_6.

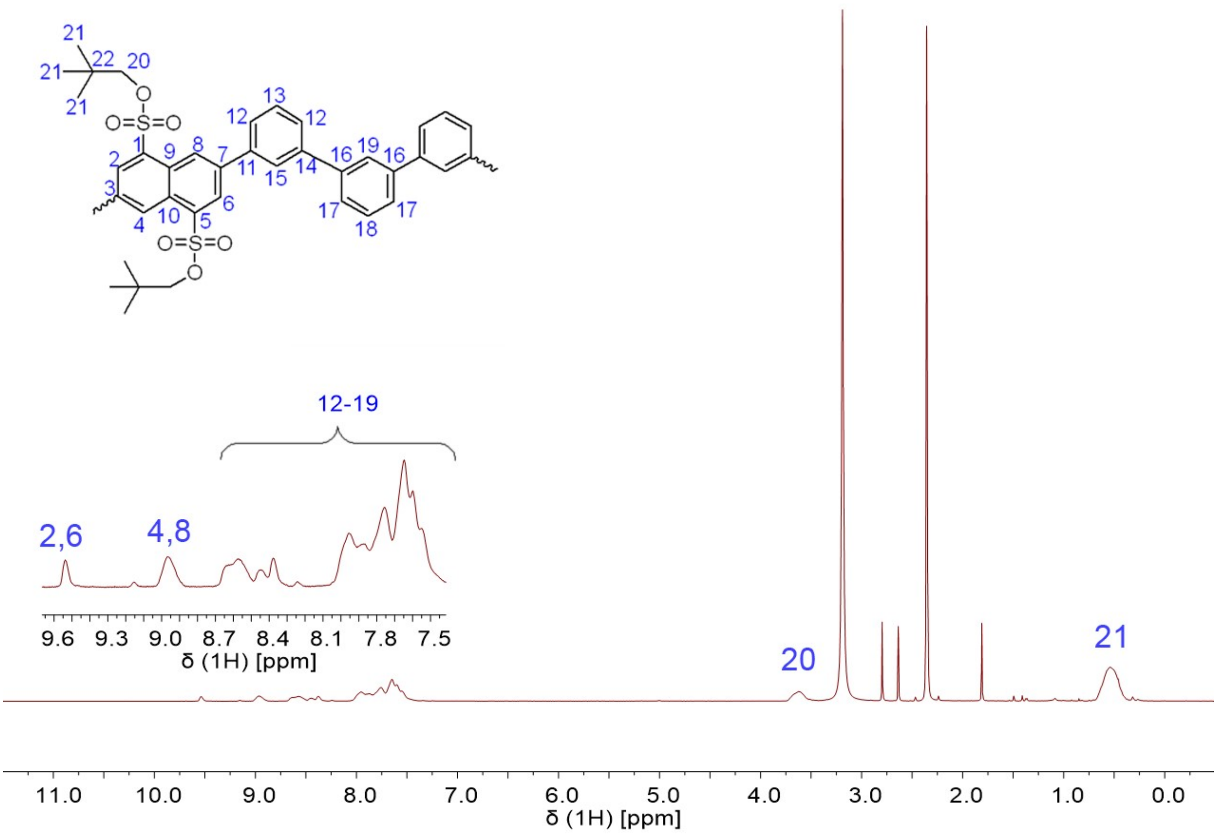

Figure S15. ^{1}H NMR spectra of polymer (**P9**) in DMSO-d_6.

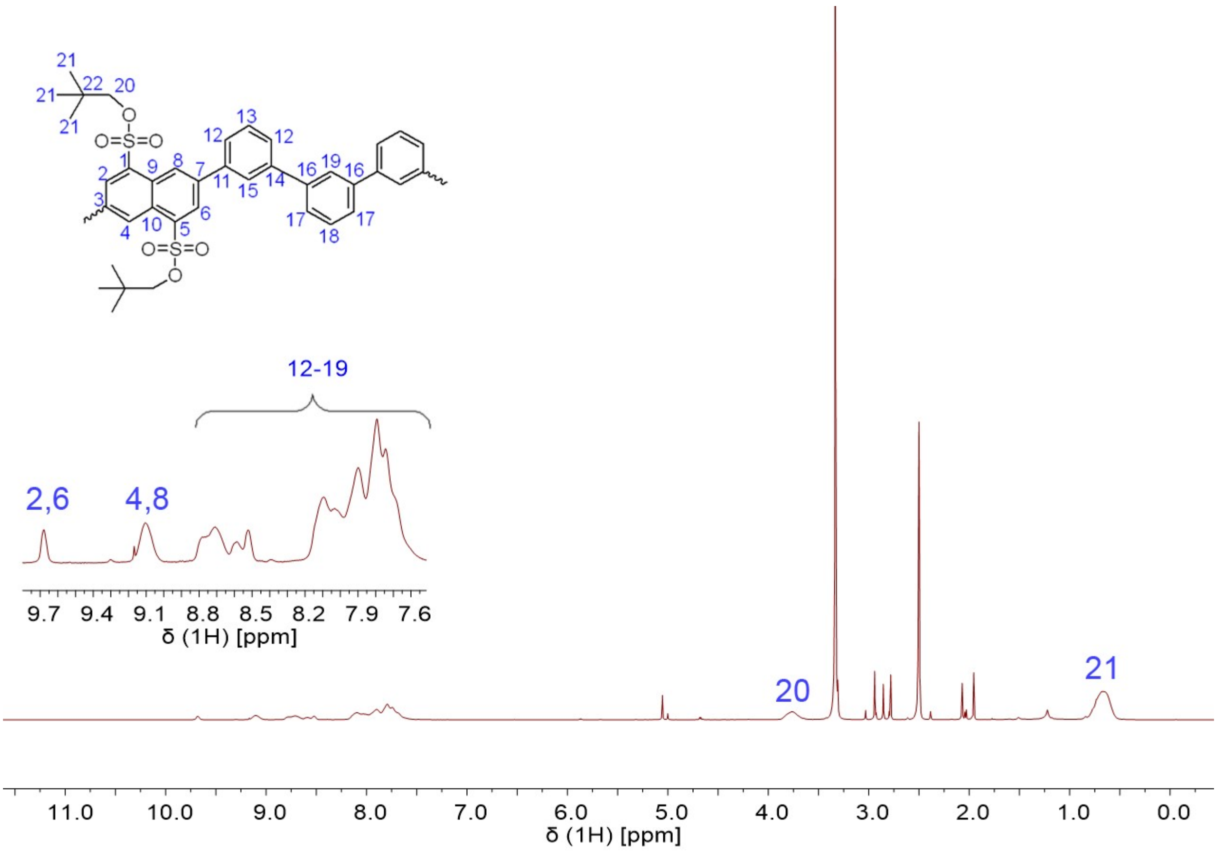

Figure S16. ^{1}H NMR spectra of polymer (**P10**) in DMSO-d_6.

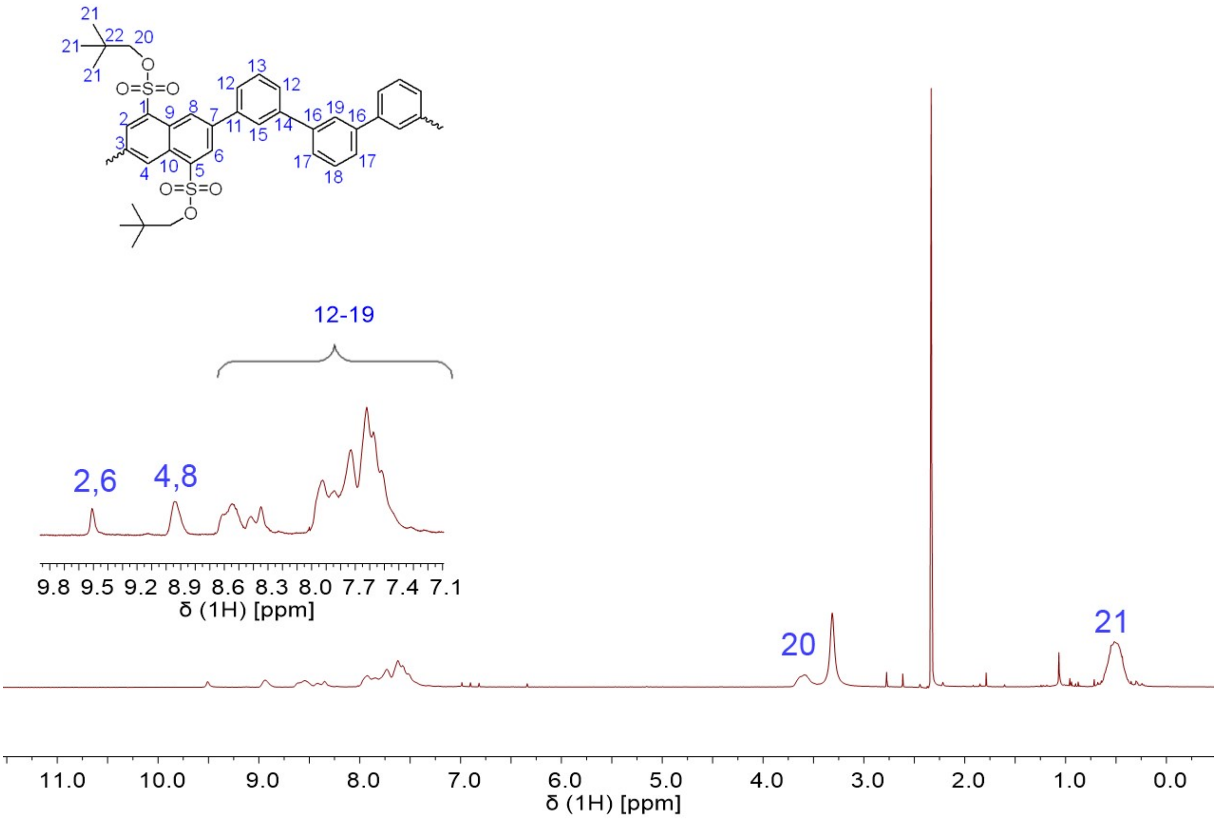

Figure S17. ^{1}H NMR spectra of polymer (**P11**) in DMSO-d_6.

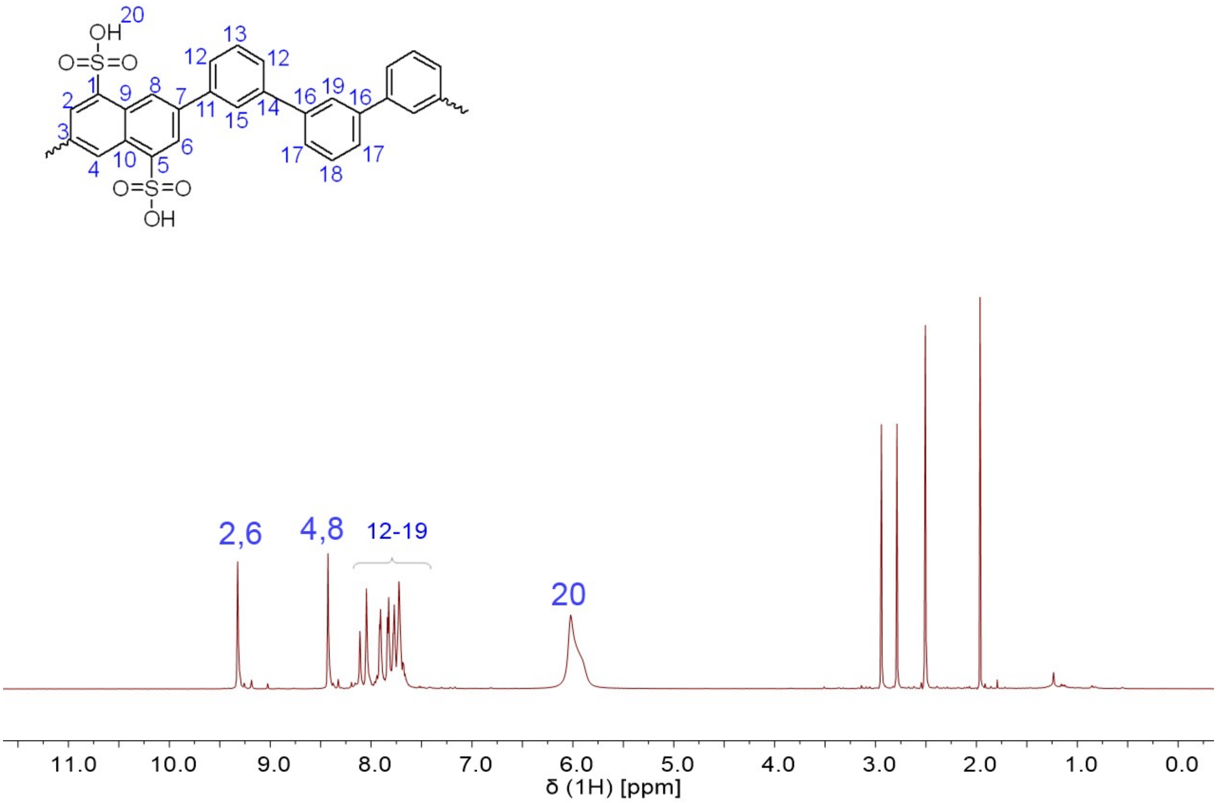

Figure S18. ^{1}H NMR spectra of the deprotected polymer (**P9**) in DMSO-d_6.

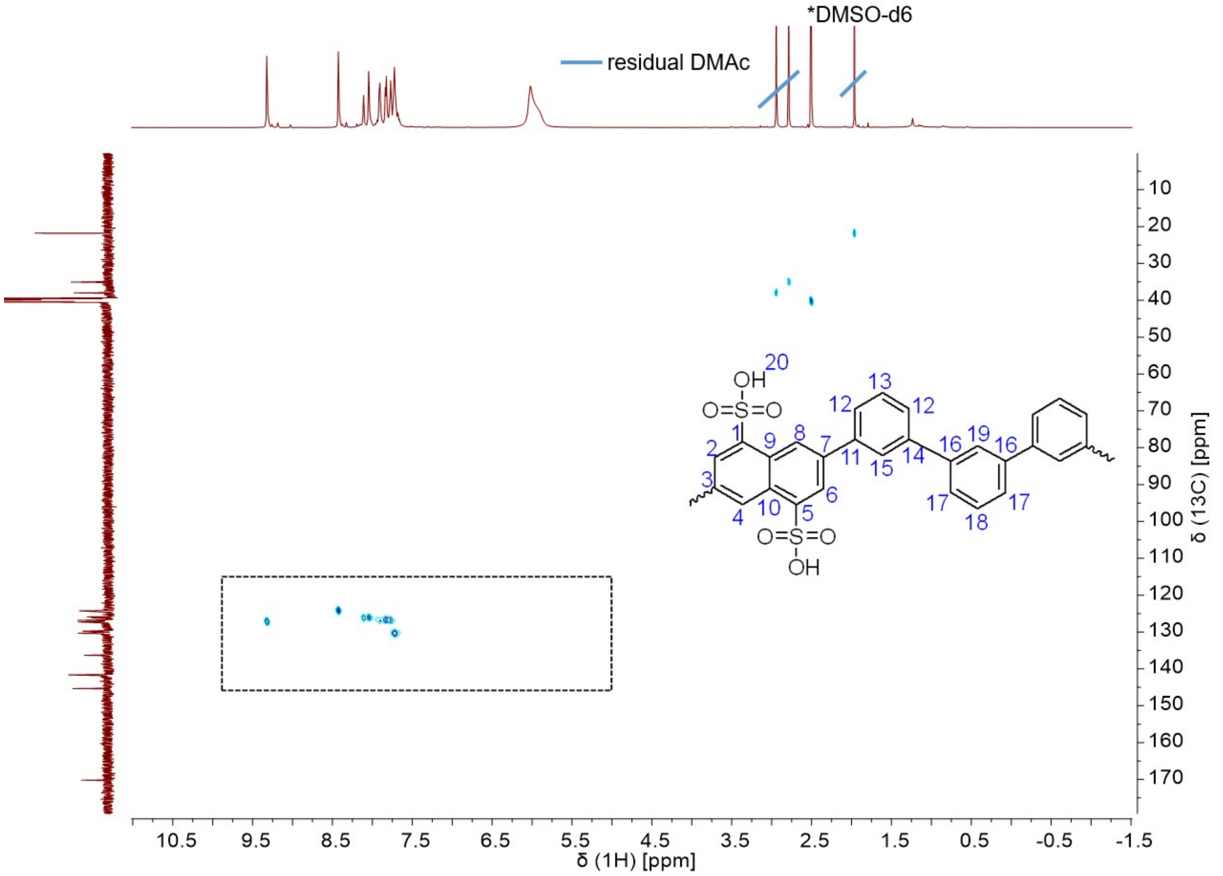

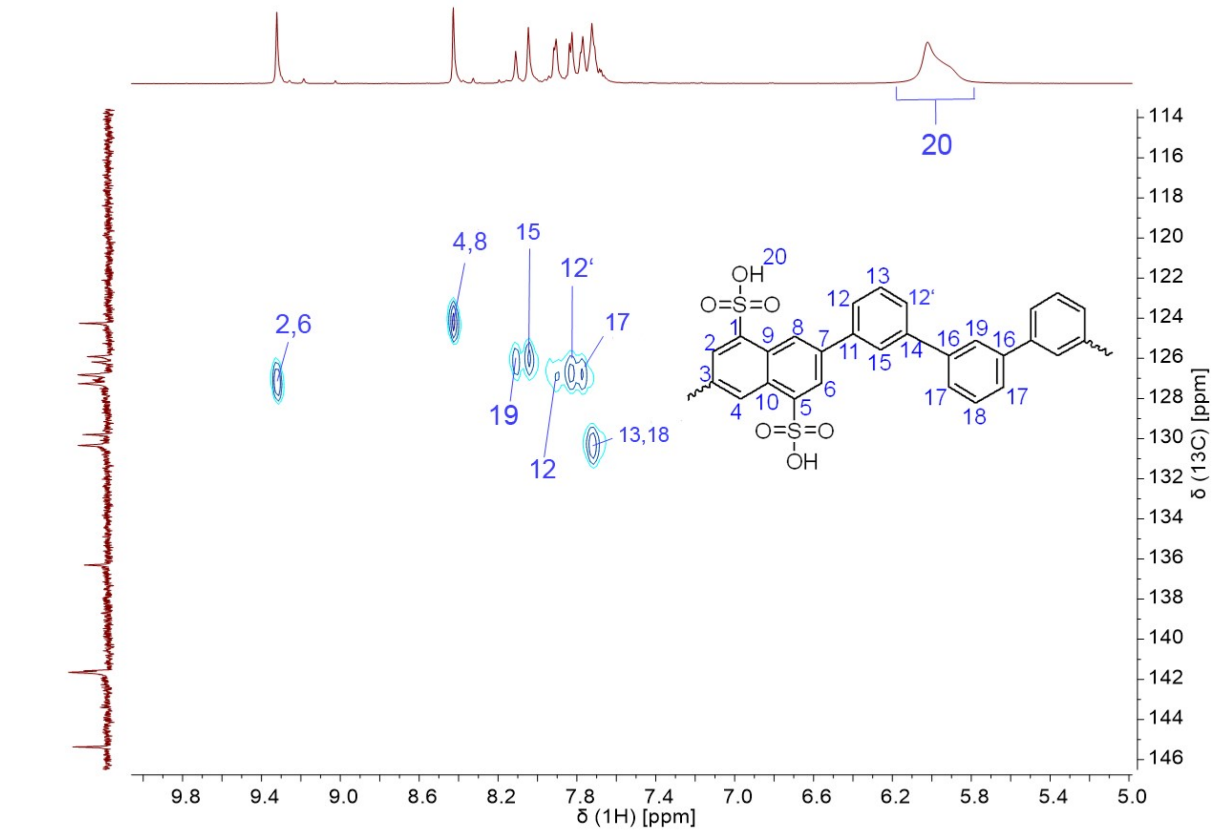

Figure S19. HSQC spectra of the deprotected polymer (**P9**) in DMSO-d_6.

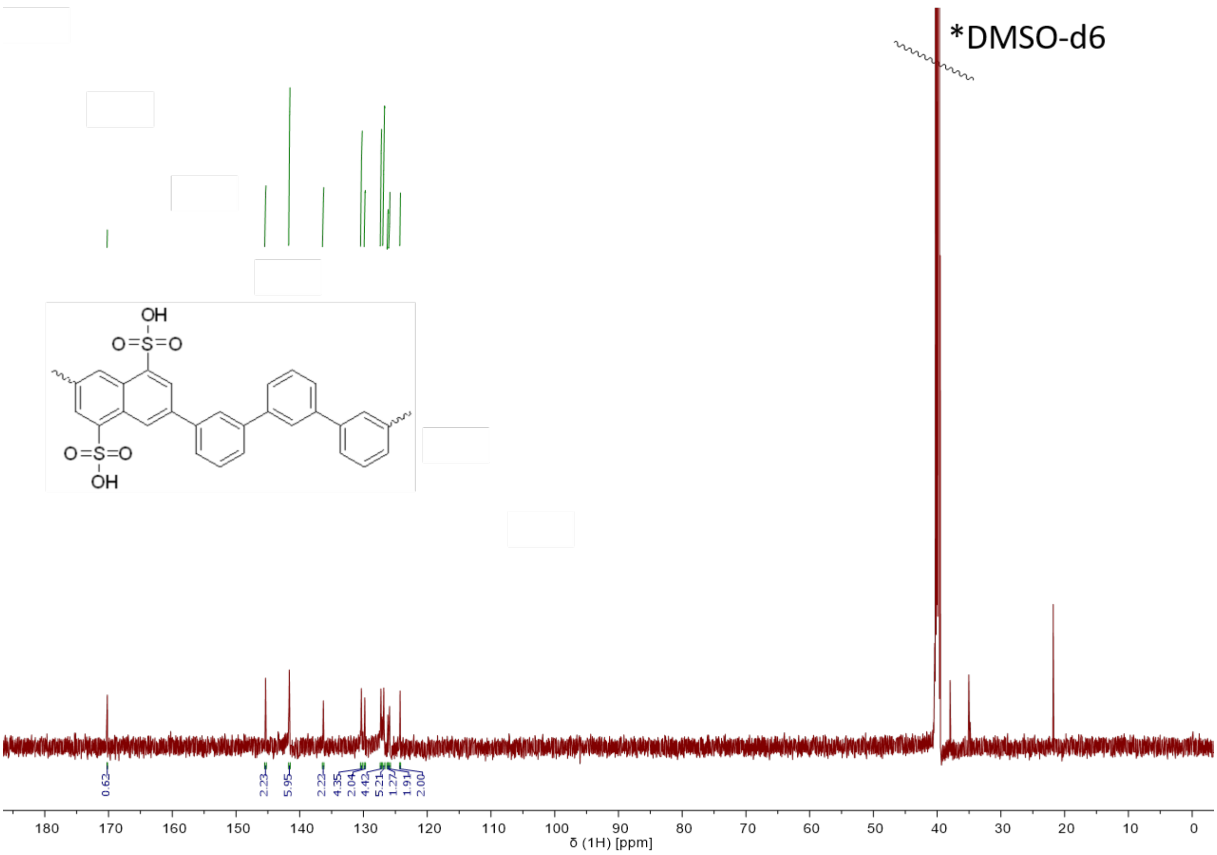

Figure S20. ^{13}C NMR spectra of the deprotected polymer (**P9**) in DMSO-d$_6$.

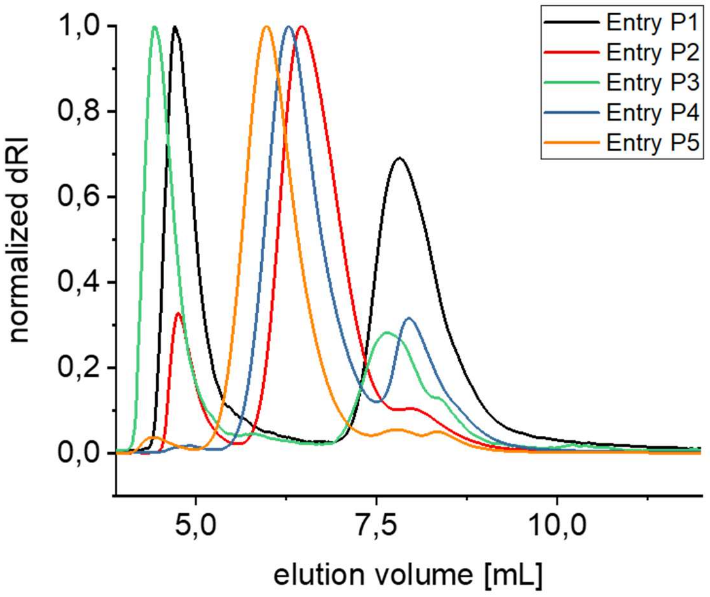

Figure S21. SEC curves of P(AA-NP-*m*P), entries **P1-5**.

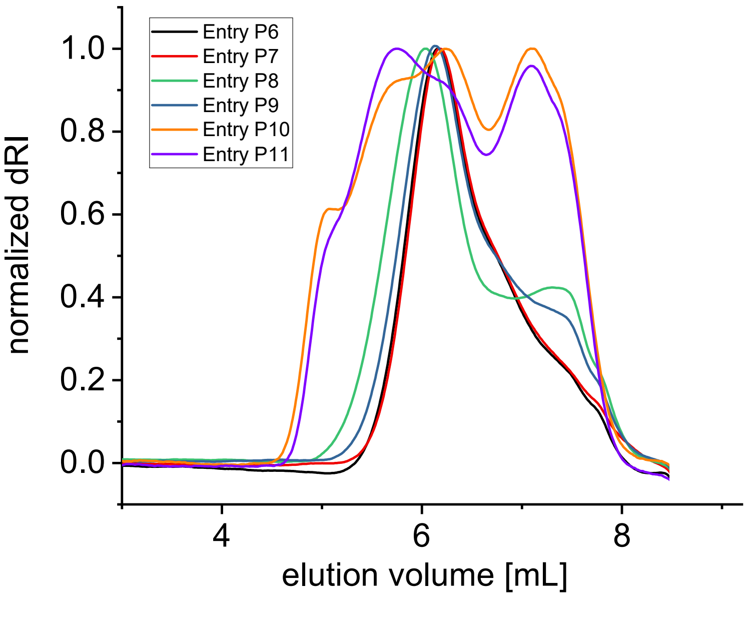

Figure S22. SEC curves of P(AA-NP-*m*TP), entries **P6-11**.

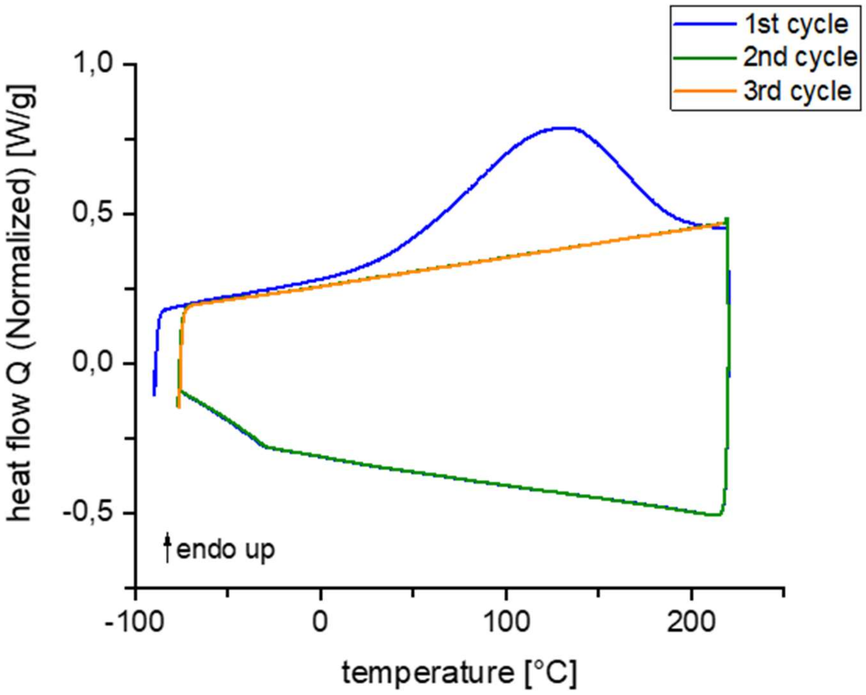

Figure S23. Differential scanning calorimetry (DSC) curve of P(AA-*alt*-*m*TP).

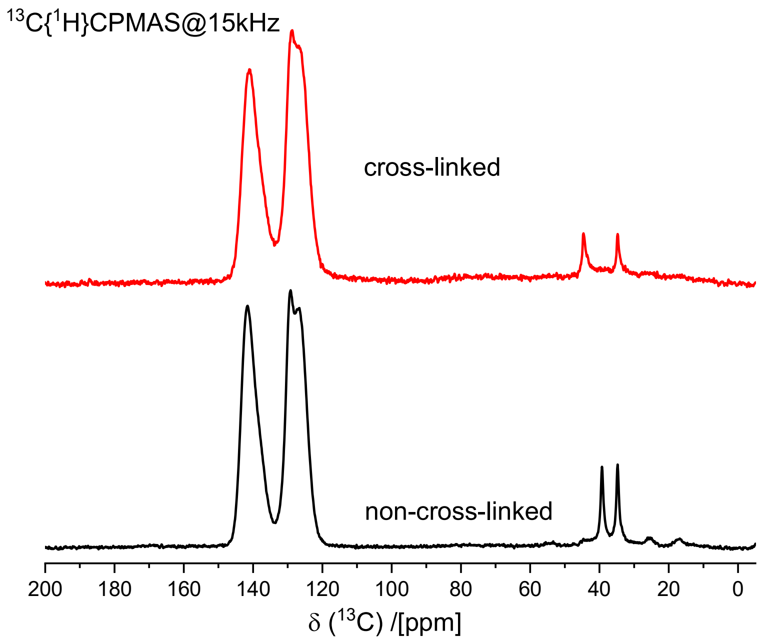

Figure S24. Solid state NMR of cross-linked and non-cross-linked P(AA-*alt*-*m*TP).

4. References

(1) Qiu, Z.-L.; Chen, D.; Deng, Z.; Chu, K.-S.; Tan, Y.-Z.; Zhu, J. Isolation of a Carbon Nanohoop with Möbius Topology. *Sci. China Chem.* **2021**, *64* (6), 1004–1008. https://doi.org/10.1007/s11426-021-9981-3.

(2) Hohenberg, P.; Kohn, W. Inhomogeneous Electron Gas. *Phys. Rev.* **1964**, *136* (3B), B864–B871. https://doi.org/10.1103/PhysRev.136.B864.

(3) Kohn, W.; Sham, L. J. Self-Consistent Equations Including Exchange and Correlation Effects. *Phys. Rev.* **1965**, *140* (4A), A1133–A1138. https://doi.org/10.1103/PhysRev.140.A1133.

(4) Becke, A. D. Density-Functional Exchange-Energy Approximation with Correct Asymptotic Behavior. *Phys. Rev. A* **1988**, *38* (6), 3098–3100. https://doi.org/10.1103/PhysRevA.38.3098.

(5) Lee, C.; Yang, W.; Parr, R. G. Development of the Colle-Salvetti Correlation-Energy Formula into a Functional of the Electron Density. *Phys. Rev. B* **1988**, *37* (2), 785–789. https://doi.org/10.1103/PhysRevB.37.785.

(6) Becke, A. D. Density-functional Thermochemistry. III. The Role of Exact Exchange. *The Journal of Chemical Physics* **1993**, *98* (7), 5648–5652. https://doi.org/10.1063/1.464913.

(7) Stephens, P. J.; Devlin, F. J.; Chabalowski, C. F.; Frisch, M. J. Ab Initio Calculation of Vibrational Absorption and Circular Dichroism Spectra Using Density Functional Force Fields. *J. Phys. Chem.* **1994**, *98* (45), 11623–11627. https://doi.org/10.1021/j100096a001.

(8) Neese, F.; Wennmohs, F.; Becker, U.; Riplinger, C. The ORCA Quantum Chemistry Program Package. *The Journal of Chemical Physics* **2020**, *152* (22), 224108. https://doi.org/10.1063/5.0004608.

(9) Neese, F. Software Update: The ORCA Program System—Version 5.0. *WIREs Computational Molecular Science* **2022**, *12* (5), e1606. https://doi.org/10.1002/wcms.1606.

(10) Weigend, F.; Ahlrichs, R. Balanced Basis Sets of Split Valence, Triple Zeta Valence and Quadruple Zeta Valence Quality for H to Rn: Design and Assessment of Accuracy. *Phys. Chem. Chem. Phys.* **2005**, *7* (18), 3297–3305. https://doi.org/10.1039/B508541A.

(11) Grimme, S.; Antony, J.; Ehrlich, S.; Krieg, H. A Consistent and Accurate Ab Initio Parametrization of Density Functional Dispersion Correction (DFT-D) for the 94 Elements H-Pu. *The Journal of Chemical Physics* **2010**, *132* (15), 154104. https://doi.org/10.1063/1.3382344.

(12) Grimme, S.; Ehrlich, S.; Goerigk, L. Effect of the Damping Function in Dispersion Corrected Density Functional Theory. *Journal of Computational Chemistry* **2011**, *32* (7), 1456–1465. https://doi.org/10.1002/jcc.21759.

(13) Barone, V.; Cossi, M. Quantum Calculation of Molecular Energies and Energy Gradients in Solution by a Conductor Solvent Model. *J. Phys. Chem. A* **1998**, *102* (11), 1995–2001. https://doi.org/10.1021/jp9716997.

(14) Di Vona et al. - 2009 - Analysis of Temperature-Promoted and Solvent-Assis.Pdf. https://pubs.acs.org/doi/pdf/10.1021/jp9006679 (accessed 2021-01-20).

(15) Soboleva, T.; Xie, Z.; Shi, Z.; Tsang, E.; Navessin, T.; Holdcroft, S. Investigation of the Through-Plane Impedance Technique for Evaluation of Anisotropy of Proton Conducting Polymer Membranes. *Journal of Electroanalytical Chemistry* **2008**, *622* (2), 145–152. https://doi.org/10.1016/j.jelechem.2008.05.017.

(16) Beyer, O.; Homburg, T.; Albat, M.; Stock, N.; Lüning, U. Synthesis of Phosphonosulfonic Acid Building Blocks as Linkers for Coordination Polymers. *New J. Chem.* **2017**, *41* (17), 8870–8876. https://doi.org/10.1039/C7NJ01697B.

(17) Blank, H. U. Process for the Preparation of Sulfonic Acid Chlorides. US4166070 (A) Abstract of corresponding document: FR2360568 (A1), August 28, 1979. https://worldwide.espacenet.com/publicationDetails/biblio?FT=D&date=19790828&DB=&locale=en_EP&CC=US&NR=4166070A&KC=A&ND=4 (accessed 2018-04-25).
